# Encapsulation of Transketolase into *In Vitro*-Assembled Protein Nanocompartments Improves Thermal Stability

**DOI:** 10.1021/acsabm.3c01153

**Published:** 2024-06-05

**Authors:** Alexander Van de Steen, Henry C. Wilkinson, Paul A. Dalby, Stefanie Frank

**Affiliations:** Department of Biochemical Engineering, University College London, Bernard Katz Building, Gower Street, London WC1E 6BT, U.K.

**Keywords:** encapsulin, nanocompartment, *in vitro* assembly, stability, protein, transketolase, cargo loading

## Abstract

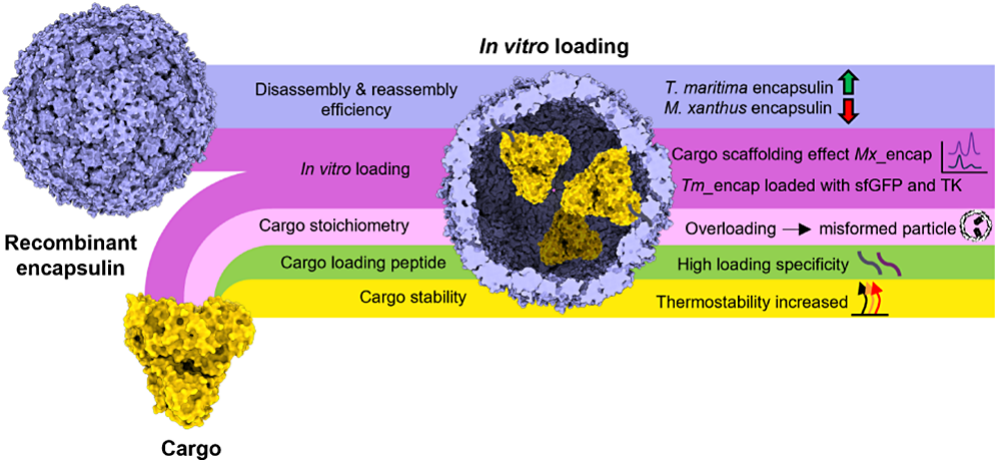

Protein compartments offer definitive structures with a large potential design space that are of particular interest for green chemistry and therapeutic applications. One family of protein compartments, encapsulins, are simple prokaryotic nanocompartments that self-assemble from a single monomer into selectively permeable cages of between 18 and 42 nm. Over the past decade, encapsulins have been developed for a diverse application portfolio utilizing their defined cargo loading mechanisms and repetitive surface display. Although it has been demonstrated that encapsulation of non-native cargo proteins provides protection from protease activity, the thermal effects arising from enclosing cargo within encapsulins remain poorly understood. This study aimed to establish a methodology for loading a reporter protein into thermostable encapsulins to determine the resulting stability change of the cargo. Building on previous *in vitro* reassembly studies, we first investigated the effectiveness of *in vitro* reassembly and cargo-loading of two size classes of encapsulins *Thermotoga maritimaT* = 1 and *Myxococcus xanthusT* = 3, using superfolder Green Fluorescent Protein. We show that the empty *T. maritima* capsid reassembles with higher yield than the *M. xanthus* capsid and that *in vitro* loading promotes the formation of the *M. xanthusT* = 3 capsid form over the *T* = 1 form, while overloading with cargo results in malformed *T. maritimaT* = 1 encapsulins. For the stability study, a Förster resonance energy transfer (FRET)-probed industrially relevant enzyme cargo, transketolase, was then loaded into the *T. maritima* encapsulin. Our results show that site-specific orthogonal FRET labels can reveal changes in thermal unfolding of encapsulated cargo, suggesting that *in vitro* loading of transketolase into the *T. maritimaT* = 1 encapsulin shell increases the thermal stability of the enzyme. This work supports the move toward fully harnessing structural, spatial, and functional control of *in vitro* assembled encapsulins with applications in cargo stabilization.

## Introduction

Protein compartments are of particular interest for green chemistry and for therapeutic applications due to their intrinsic properties: self-assembling, defined, closed structures, ranging across the nanometer length scale, composed of repeating subunits capable of selective packaging of biomacromolecules.^1,2^ Extensive engineering efforts have been applied to an array of compartments, with the common goal of a modular chassis with discrete commutable parts. Modularity holds the promise of chimeric vaccines and targeted drug delivery systems for the rapid development of economical vaccines and targeted medicine.^1,3^ Industrial applications include semicontinuous processes and orthogonal cellular nanoreactors.^4,5^

*In vitro* loading from a defined environment into protein compartments is a vital aspect for full exploitation of such compartments for use as a platform technology. Virus-like particles (VLPs) have been demonstrated to load a variety of cargos via electrostatic interactions, engineering of the luminal surface^6^ or via direct genetic fusion for proteinaceous cargos.^7^ Most targeted drug delivery systems have focused on cancer therapeutic applications delivering small cytotoxic prodrugs/drugs to the local tumor environment, either passively via enhanced permeability and retention (EPR) effect or targeted by surface modifications. Furthermore, targeted mRNA cargos have also been demonstrated in VLPs utilizing the native luminal affinity to nucleic acids or with peptide sequences with specific affinity for mRNA stem loop sequences loaded *in vitro*.^8^ However, potential peptide cargo loading within protein compartments is limited by expensive conjugation strategies, low-throughput genetic insertions and fusions, or low-specificity passive uptake during reassembly.^3,9^

Encapsulin nanocompartments are an attractive family for protein compartment engineering. Composed of a single self-assembling capsid protein within the HK97 protein superfold, encapsulins are widespread through bacteria and archaea kingdoms,^10,11^ usually displaying high thermal,^12^ mechanical,^13^ and pH stability.^14,15^ Three capsid icosahedral size classes have so far been characterized by triangulation number (*T* number), *T* = 1 (60 monomers, 18–24 nm),^14,16,17^*T* = 3 (180 monomers, 32 nm),^18,19^ and *T* = 4 (240 monomers, 42 nm).^20^ Larger dsDNA HK97 phage capsids use accessory scaffolding proteins to achieve *T* = 7 and larger sizes.^21^ Native encapsulin functions range from mitigating oxidative stress,^22^ iron sequestration,^18^ sulfur metabolism^16^ to sequestering energic anammox intermediaries.^10^ Function is directed through protein cargo loading via a conserved short cargo loading peptide (CLP) commonly seen as an N- or C-terminal fusion. The role that cargo loading plays on encapsulin capsid assembly and size determination is not yet well understood. A potential scaffolding role of cargo protein has been suggested for the *Myxococcus xanthus* encapsulin, capable of forming both *T* = 1 and *T* = 3 capsids. When expressed *in vivo* with native cargo, the ratio of *T* = 3 capsids has been reported to be increased.^23^ The *Quasibacillus thermotolerans* encapsulin, on the other hand, does not seem to require cargo protein loading for full *T* = 4 conformation.^20^

Encapsulin have demonstrated applications as both cellular nanoreactors^24−27^ and modular drug delivery systems^27−29^ through loading of non-native protein cargo appended with a CLP sequence. To load *in vitro*, capsids must first be assembled *in vivo*, purified from the cell lysate, and then disassembled and reassembled *in vitro*. This reassembly has been well established for *T* = 1 encapsulins via extreme pH and high guanidium hydrochloride concentrations^12,15,30^ and has been shown at specific concentrations of GuHCl for *T* = 3 *M. xanthus* and *T* = 4 *Q. thermotolerans* encapsulins.^12^ Reassembly has been coupled to *in vitro* loading of fluorescent proteins when incubated or dialyzed in the reassembly conditions. However, low loading stoichiometries have been observed, attributed to the oligomeric difference of the native to fluorescent cargos.^31,32^ Cargo loading, however, has also been demonstrated with other biomolecules. Recently, dual *in vivo* loading of specific protein and cellular mRNA was demonstrated by insertion of a lysine-rich peptide to the luminal surface of the encapsulin monomer alongside using the CLP.^33^ Stimulated encapsulin disassembly and drug release remain issues for encapsulin-based therapeutics. Drug delivery applications and release of cargo, however, will require mild disassembly conditions such as action of cellular proteases,^34^ low pH,^35^ or laser/sonically stimulated disruption.^36^

Equivalent to viral delivery, larger compartments are expected to allow for a greater cargo loading capacity. So far, there have been limited *in vitro* loading attempts of larger encapsulin variants. Recently, the *Q. thermotoleransT* = 4 encapsulin was demonstrated to be capable of mNeon cargo loading. An insertion of a pH responsive helix in the E loop allowed triggered disassembly at pH 6.0 and reassembly to a uniform *T* = 4 capsid with increasing pH.^37^

Encapsulation of therapeutic molecules is a common strategy to increase specific drug stabilities that are important for clinical use. Encapsulins have demonstrated nonspecific protease resistance,^15,38^ which is conveyed to encapsulated cargo. It is well known that enzymatic immobilization strategies enhance stability via concentration-related effects.^4^ However, how encapsulation affects the thermal stability of cargo within the encapsulin beyond functional enzymatic reactions has not been probed, to the best of our knowledge. Here, we first evaluate the efficiency of *in vitro* reassembly in common denaturants based on previous studies^12,15^ and the effect of *in vitro* cargo loading on *T. maritima* and *M. xanthus* capsid assembly. We then demonstrate *in vitro* loading with transketolase and show encapsulated enzyme activity before using a click-chemistry FRET pair to probe thermal stability perturbations resulting from encapsulation. We show that the compact nature of encapsulation significantly alters the transketolase FRET emission profile, where a 15 °C increase in *T*_m_ was observed between encapsulated and free transketolase detected by discrete transitions in the FRET donor emission signal. The characterization of *in vitro* loading allowed us to quantify the stability gain of encapsulin cargo loading, vital for drug delivery and nanoreactor applications.

## Results

**Figure 1:**
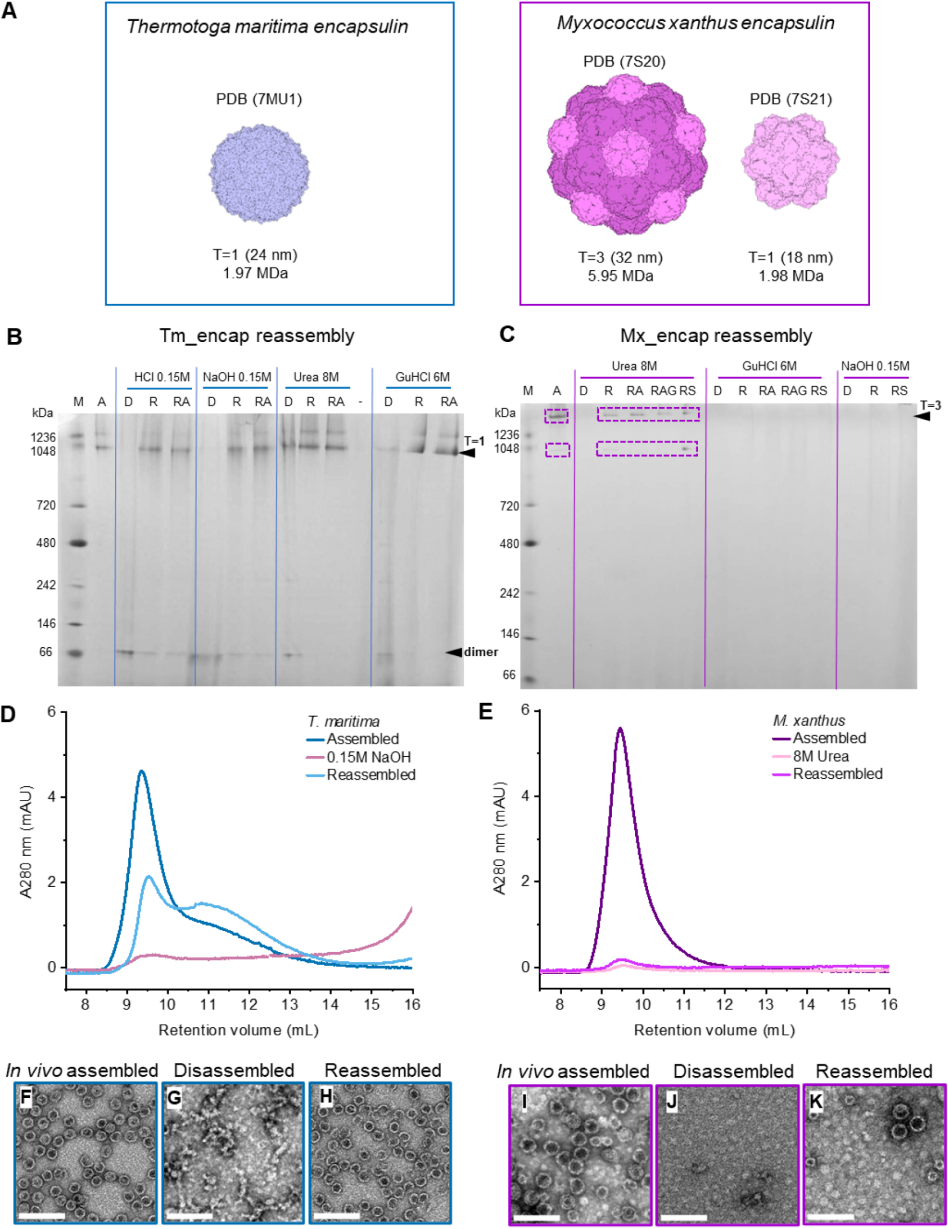
*In vitro* reassembly of *T. maritima* and *M. xanthus* encapsulins. (A) Capsid structures and sizes of *T. maritimaT* = 1 encapsulin and *M. xanthusT* = 3 and *T* = 1 encapsulins. (B, C) BN-PAGE of Tm_encap and Mx_encap reassembly reaction conditions (not SEC purified). A = assembled, D = denatured, R = reassembled in reassembly buffer, RA = reassembly buffer +250 mM l-arginine, RAG = reassembly buffer + 250 mM l-arginine + 20% v/v glycerol, RS = reassembly buffer + 0.5 M sorbitol. (D, E) SEC A_280_ profiles of Tm_encap and Mx_encap, assembled, disassembled, and reassembled with denaturation conditions indicated. (F–H) TEM micrographs of Tm_encap, *in vivo* assembled, disassembled (0.15 M NaOH), and reassembled, images from pooled SEC fractions 8.5–14 mL. Scale bar = 100 nm. (I–K) TEM micrographs of Mx_encap, *in vivo* assembled, disassembled (8 M urea), and reassembled, images from pooled SEC fractions 8.5–12 mL. Scale bar = 100 nm.

We first report our findings on *in vitro* sfGFP cargo loading into two recombinantly produced empty encapsulins from *T. maritima* and *M. xanthus*, followed by *in vitro* loading of the enzyme transketolase (TK) and thermal stability analysis. Throughout the paper, we refer to the quaternary structures of the empty *T. maritima* and *M. xanthus* encapsulin shells as Tm_encap and Mx_encap, respectively. Tm_encap assembles into *T* = 1 capsids (1.97 MDa), and Mx_encap assembles into two sizes with triangulation numbers *T* = 1 (1.98 MDa) and *T* = 3 (5.95 MDa) (Figure 1A).

### Capsid Disassembly and Reassembly Conditions

Previous studies have reported *in vitro* encapsulin disassembly and reassembly under a range of conditions (Table 1). Based on this work, we initially tested high concentrations of denaturants, 6 M guanidinium hydrochloride (GuHCl) and 8 M urea in 20 mM Tris-Cl pH 7.5 and extreme pH conditions (0.15 M HCl, pH 1.0 and 0.15 M NaOH, pH 13.0). Tm_encap and Mx_encap were purified from *E. coli*, disassembled in the stated conditions, and reassembled by 10-fold dilution in reassembly buffer (0.3 M Tris-Cl pH 7.5, 0.15 M NaCl, 5 mM β-ME). For direct visualization, the samples were evaluated by Blue Native (BN)-PAGE electrophoresis. For the initial reassembly experiment, samples were not subjected to further purification with size exclusion chromatography (SEC) as the heterogenicity of samples was of interest, in particular, the appearance of protomers and intermediate assemblies. Samples were further SEC-purified for the loading capacity studies and TK experiments, as stated in the various sections. It should also be noted that encapsulins do not migrate exactly at the calculated molecular weight,^15,16,39^ but the BN-PAGE marker is still a suitable reference for high molecular weight species.

**Table 1:** Summary of the Reported Disassembly and Reassembly Conditions Used for Encapsulins^a^

encapsulin	denaturant	final monomer concentration	reassembly buffer	reassembly trigger	*in vitro* loading	source
*T. maritima*	4–6 M GuHCl	not stated	50 mM HEPES, 1 mM DTT, pH 7.4	dialysis	no loading	(12)
*M. xanthus*						
*Q. thermotolerans*						
*T. maritima*	7 M GuHCl, 1 mM DTT	8 μM	20 mM sodium phosphate, 50 mM NaCl, pH 7.4	10-fold dilution	*in vitro* loading 2:1 ratio of sfGFP:capsid monomer	(15)
	0.15 M NaOH, 1 mM DTT					
	0.15 M HCl, 1 mM DTT					
*T. maritima*	10 mM phosphate buffer, pH 2.0	0.16 μM	20 mM phosphate, pH 7.0	100-fold dilution	*in vitro* loading 1:1 ratio of gold nanoparticles:capsid	(40)
*R. jostii*	100 mM acetate buffer, pH 3.0	3 μM	100 mM sodium phosphate, 100 mM NaCl, pH 7.4	rebuffered to pH 7.4	*in vitro* loading 0.25:1 ratio of DypB native cargo:capsid monomer	(30)
*R. erythropolisN771*	6 M GuHCl, 0.5 M arginine	not stated	50 mM phosphate, 0.5 M NaCl, 1 mM DTT, pH 8.0	dialysis	no loading	(38,41)
*S. elongatusPCC 7942*	6 M GuHCl, 50 mM Tris HCl, 50 mM DTT, pH 7.4	not stated	50 mM CAPS, 0.25 M arginine, 150 mM NaCl, 20% glycerol, 10 mM DTT, pH 10.0	100-fold dilution	*in vitro* loading 10:1 ratio of native cargo:capsid monomer	(16)
*This Study*						
*T. maritima*	0.15 M NaOH	10 μM	0.3 M Tris-HCl 0.15 M NaCl, pH 7.5	10-fold dilution	*in vitro* loading of 0.2–15:1 ratio sfGFP:capsid monomer; *in vitro* loading of transketolase 1:1 ratio of TK:capsid monomer	this study
*M. xanthus*	8 M Urea, pH 7.0	10 μM	0.3 M Tris-HCl 0.15 M NaCl, pH 7.5	10-fold dilution	*in vitro* loading of 0.2–10:1 ratio sfGFP:capsid monomer	this study

a *In vitro* loading of cargo proteins is listed with molar ratio of cargo to encapsulin.

### Tm_encap Reassembles after pH Denaturation

When disassembly of Tm_encap was tested using high concentration denaturants, specifically 6 M GuHCl as previously reported^12^ and 8 M urea, the *T* = 1 encapsulin band (migrating at the 1048 kDa marker) was reduced but did not fully disappear in the BN-PAGE (Figure 1B), indicating that the capsids had not completely disassembled. Under extreme pH conditions of 0.15 M NaOH (pH 13.0) and 0.15 M HCl (pH 1.0), the *T* = 1 capsid band disappeared on BN-PAGE, and a smaller band became visible near the 66 kDa marker, indicating the presence of dimers with a predicted molecular weight of ∼65 kDa (Figure 1B). After reassembly, a strong *T* = 1 capsid band reappeared for both pH conditions suggesting reassembly. The dimer band was also seen under denaturing conditions (6 M GuHCl and 8 M Urea) and is thought to be a key intermediate in *T* = 1 capsid assembly.^12,13^ The larger band observed above the *T* = 1-associated band in Native PAGE is commonly seen by us and others in Native PAGE.^12^ It is thought to be due to encapsulin aggregates,^12^ rather than impurity as indicated by the absence of contaminating proteins after Strep-tag purification (Figure S1). Alternatively, it could result from isoforms, such as incomplete structures like multimers of late-stage assembly intermediates.^18^

### Mx_encap Reassembly Is Dependent on Denaturant Choice in the Disassembly Step

Mx_encap was also reported to disassemble and reassemble under denaturing conditions with GuHCl.^12^ When we tested 6 M GuHCl, complete disassembly of the Mx_encap (loss of both *T* = 3 and *T* = 1 related BN-PAGE bands) was seen, but reassembly was not observed (Figure 1C). Similarly, basic (0.15 M NaOH, pH 13.0) treatment showed full disassembly of the Mx_encap but no reassembly, which was also reported by Boyton et al.^12^ Acidic treatment (0.15 M HCl, pH 1.0) led to irreversible capsid aggregation (not shown). Only 8 M urea treatment and subsequent dilution in reassembly buffer produced both *T* = 3 and *T* = 1 bands on BN-PAGE (Figure 1C) and evidence of reassembled capsids of both sizes in the TEM (Figure 1K). These findings indicate that in contrast to Tm_encap, extreme pH conditions lead to disassembly products that cannot effectively regain their native conformation upon dilution. It is unclear why the urea denaturant condition allowed for reassembly whereas GuHCl did not, despite having been reported as suitable for reassembly.^12^ GuHCl and urea have been shown to have different effects on protein unfolding in various transition states. Reassembly efficiency is likely related to these disassembly states. To achieve optimal reassembly efficiency, the chaotrope concentration may need to be optimized. The use of popular protein renaturation additives such as arginine, sorbitol, and glycerol did not increase reassembly (Figure 1C).

**Figure 2:**
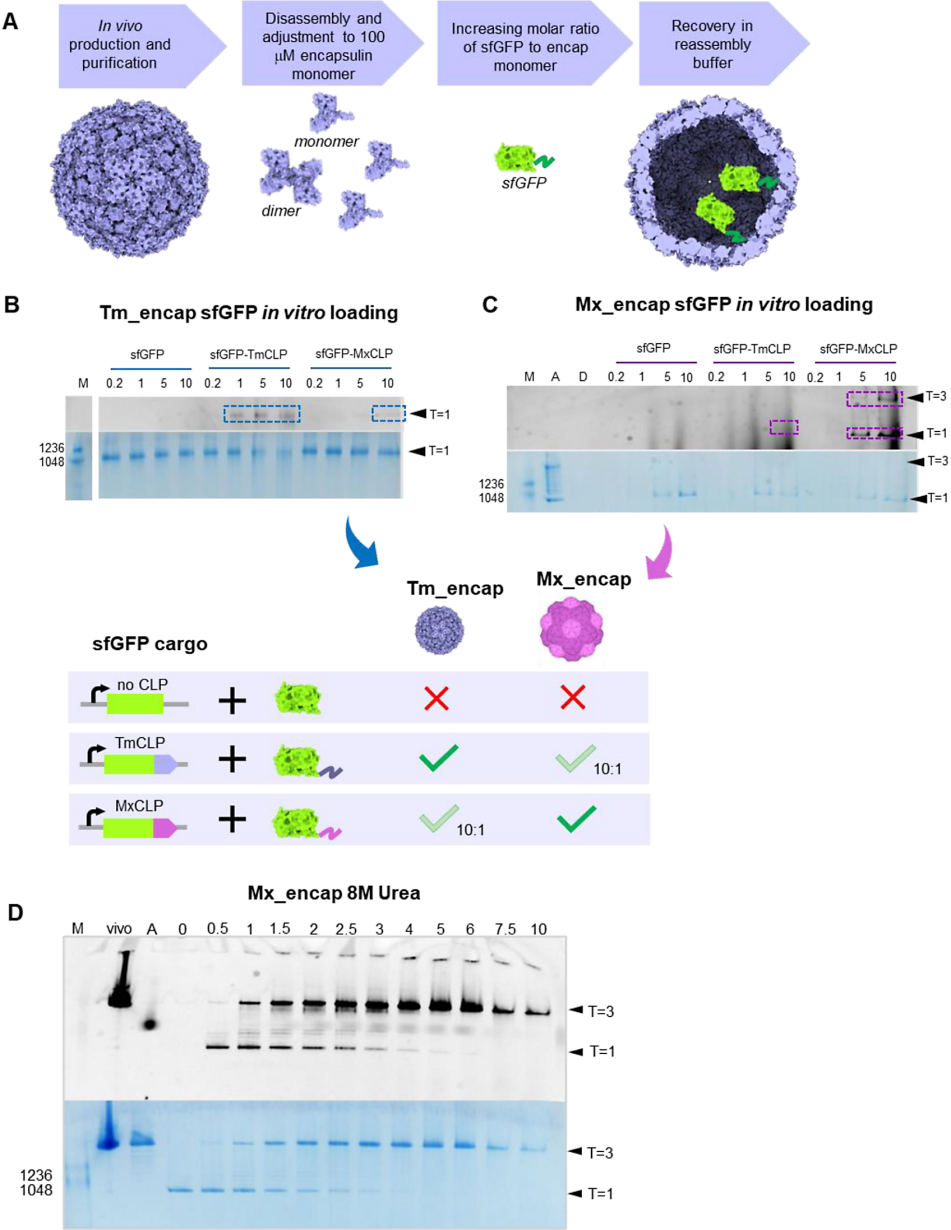
*In vitro* sfGFP cargo loading specificity and scaffolding effect. (A) Workflow of *in vitro* sfGFP cargo loading method. Reassembly of encapsulin proteins was initiated by 10 times dilution of denaturant condition with reassembly buffer (0.3 M Tris-Cl pH 7.5, 0.15 M NaCl) to a final monomer concentration of 10 μM. (B, C) sfGFP loading into the Tm_encap (B) and sfGFP loading into the Mx_encap (C). Molar ratio of sfGFP to encapsulin monomer 0.2, 1, 5, and 10 to 1, respectively. M = molecular weight marker. Top black and white image shows fluorescence signal of sfGFP. Bottom image shows Coomassie-stained BN-PAGE gel. Full BN-PAGE is shown in Figure S2. (D) sfGFP cargo loading into Mx_encap (disassembled in 8 M urea) at increasing concentrations of sfGFP showing a decrease of assembly with high sfGFP concentration. Top black and white image shows fluorescence signal of sfGFP, bottom image shows Coomassie-stained BN-PAGE gel. M = molecular weight marker, vivo = *in vivo*-loaded encapsulins, A = assembled (before denaturation), and numbers in lanes indicate molar ratio of sfGFP to encapsulin monomer.

Furthermore, both our observations and those from other studies indicate variability in the *T* = 3 to *T* = 1 ratios of recombinant empty Mx_encap compartment preparations from *E. coli*, with *T* = 1 reported to range between 20% and 36%.^18,23^ Additionally, a range of intermediate sizes between *T* = 1 and *T* = 3 were observed in our study (Figure S3) and other work.^12^ The purified encapsulin sample utilized for our disassembly/reassembly study predominantly contained *T* = 3 capsids (Figure 1C), whereas samples prepared for the sfGFP loading experiments showed a higher fraction of *T* = 1 capsids (Figure 2C). While the molecular mechanism behind this variation is not fully understood, we demonstrate further on in this paper that the Mx_encap capsid size can consistently be shifted toward *T* = 3 by *in vitro* cargo loading, akin to what has been observed *in vivo*.^23,42^

### Reassembly Efficiency Is Higher for Tm_encap Compared to Mx_encap

The denaturant conditions with the highest reassembly efficiency judged visually from the BN-PAGE, 0.15 M NaOH and 8 M urea for Tm_encap and Mx_encap, respectively, were selected for further characterization, using size exclusion chromatography (SEC) and transmission electron microscopy (TEM) (Figure 1D–K). After reassembly, SEC profiles of Tm_encap showed an increase in shoulder peak at 11 mL compared with before disassembly (Figure 1D). This could be due to nonregular, partially closed capsids eluting later than the intact capsids. Taking the peak area between the retention volumes corresponding to higher-order structures of Tm_encap (8–14 mL), we determined the recovery to be 82%. Transmission electron microscopy (TEM) of the pooled fractions revealed reassembly into *T* = 1 particles, resembling the *in vivo* assembled structures, alongside a few open particles likely originating from the shoulder fraction in the SEC profile (Figure 1F–H). In contrast, the reassembly efficiency of *M. xanthus* encapsulin was low (Figure 1E), evidenced by a SEC peak area of 4.8% calculated from the area between 8.5 and 12 mL. Additionally, the number of particles observed in TEM images from this peak was reduced compared to that of Mx_encap before disassembly (Figure 1I–K).

### Evidence for Cargo Loading Peptide Dependence

Initially, exploratory experiments were conducted to test cargo loading peptide dependence when loading a protein (sfGFP) *in vitro*. When the transketolase loading described later in this paper was performed, the results were found to be reproducible. *In vitro* loading of purified sfGFP into disassembled and reassembled Tm_encap and Mx_encap capsids was carried out with and without the fusion of the known cargo loading peptides (CLPs)^15,18^ from *T. maritima* (TmCLP) and *M. xanthus* (MxCLP) to the C terminus of sfGFP. Tagged sfGFP was supplemented into the reassembly buffer at molar ratios ranging from 0.2 to 10 times (2–100 μM) sfGFP relative to the encapsulin concentration (10 μM) in the final reassembly mix (Figure 2A). Reassembled samples were directly subjected to BN-PAGE without further purification to visualize all assembly products. Evidence for sfGFP encapsulation was determined by overlaying the capsid band on the Coomassie-stained BN-PAGE gel with the fluorescence image (Figure 2B and C).

No sfGFP fluorescence was detected at the Tm_encap and Mx_encap bands in the absence of CLP, even at the highest sfGFP concentrations tested. With the sfGFP protein volume (∼25 nm^3^) approximately 100-fold smaller than the internal volume of a *T* = 1 capsid (∼3000 nm^3^), passive loading would be expected upon the structure closing, particularly at high concentrations of sfGFP. However, the lack of observable fluorescence indicates that sfGFP is excluded during assembly or that the concentration of sfGFP inside the capsid is below the detection threshold. When incubated with the matching cargo loading peptide (sfGFP-TmCLP), fluorescence was observed at the Tm_encap *T* = 1 band. Minimal cross-loading of the MxCLP-tagged sfGFP into Tm_encap was observed at high molar concentration, showing a faint fluorescence signal at the Tm_encap band at 10:1 ratio of sfGFP-MxCLP to capsid (Figures 2B and S2A). With regards to Mx_encap, sfGFP-MxCLP signal is observed at the *T* = 1 Mx_encap band, and at higher concentrations, a higher band appears suggestive of *T* = 3 capsids; however, no obvious *T* = 3 band is visible in BN-PAGE. A weak band can be seen with the non-native TmCLP at a 10:1 ratio, suggesting minimal cross loading (Figures 2C and S2B).

The data suggests that like *in vivo* loading,^15,18,20^*in vitro* loading is mediated by CLPs. Our findings indicate that the cargo is likely to be located inside the capsid, as an external association, such as the one reported for His-tagged sfGFP when mixed with fully assembled Tm_encap,^15^ would be less specifically controlled by the CLP, and one would expect to see colocalization of sfGFP without the CLP, as well as enhanced cross-loading. However, association between cargo and capsid warrants further investigation in the future, particularly with regard to compartments that are not completely closed, as described later in this work.

### Loading Capacity

**Figure 3:**
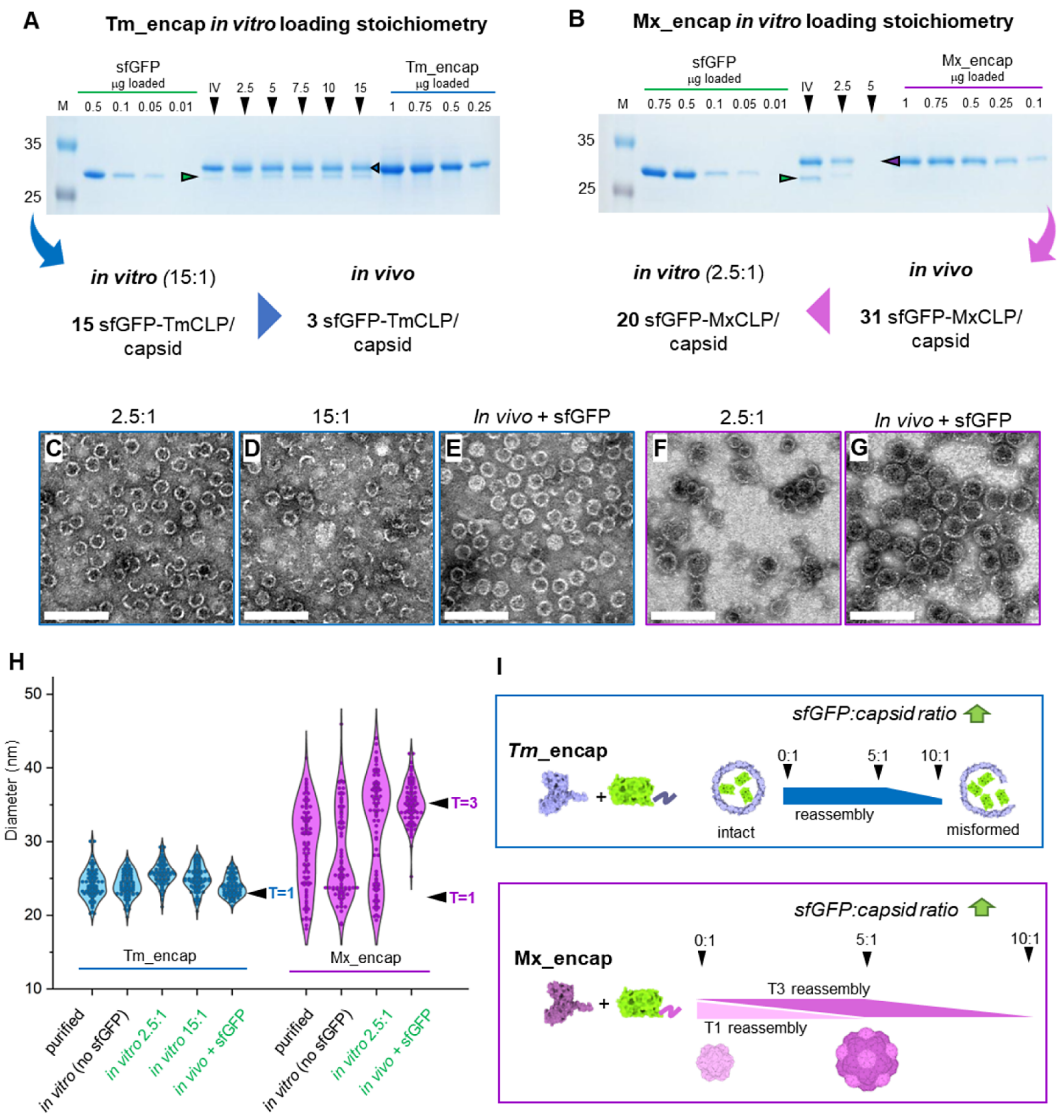
*In vitro* sfGFP cargo loading and scaffolding effect on *M. xanthus* encapsulin. All data shown are from SEC purified samples. (A, B) SDS-PAGE densitometry of *in vitro-* versus *in vivo*-loaded encapsulin. Schematic below A and B shows summary of loading capacity of *in vitro* versus *in vivo* sfGFP-loaded encapsulins. Data were derived from a single experiment. (C–G) TEM micrographs of the following: *in vitro*-loaded Tm_encap with sfGFP-TmCLP, molar ratios indicated (C, D); *in vivo*-loaded Tm_encap with sfGFP-TmCLP (E); *in vitro*-loaded Mx_encap with sfGFP-MxCLP (F); and *in vivo*-loaded Mx_encap with sfGFP-MxCLP (G). TEM scale bar = 100 nm. (H) Violin plot of TEM diameter measurement of capsids before and after *in vitro* loading of sfGFP versus *in vivo* loading. Measurements of 100 particles per sample. Associated *T* = 3 and *T* = 1 size range indicated. (I) sfGFP:capsid monomer concentration affects *in vitro* capsid assembly efficiency and capsid properties. Increasing sfGFP concentrations lead to misformed Tm_encap and higher proportion of *T* = 3 over *T* = 1 Mx_encap species (scaffolding effect), followed by a decrease in Mx_encap assembly when ratio is above 5:1.

Next, sfGFP-*in vitro*-loaded *T. maritima* and *M. xanthus* encapsulins (with corresponding CLP) were purified further by SEC to remove nonencapsulated sfGFP (for representative SEC profile see Figure S4). Loading capacity was determined via SDS-PAGE densitometry against a standard range (Figure 3A,B) and *in vitro*-loaded encapsulins compared to *in vivo*-loaded samples (*in vivo* loading is described in Materials and Methods). A loading stoichiometry of 2.94 molecules per *in vivo*-loaded Tm_encap was determined, in line with previously recorded fluorescent protein loading stoichiometries.^15,31,32^*In vitro* loading stoichiometry is positively correlated with cargo concentrations during reassembly exceeding the *in vivo* loading ratios at a 5:1 molar ratio. At 15:1 molar ratio, the loading was determined to be 15 copies of sfGFP, approximately a 5-fold increase to the *in vivo*-loaded control. The Tm_encap *T* = 1 capsid diameter measured via TEM did not shift in response to *in vivo* or *in vitro* sfGFP-TmCLP loading (Figure 3H). However, visually, more incomplete capsids were detected at 2.5:1 and were even more apparent at 15:1 molar ratios compared to *in vivo*-loaded capsids (Figure 3C–E). We hypothesize that while more copies of sfGFP may be loaded at higher molar ratios, this is at the expense of capsid closure. *In vitro*-loaded Mx_encap showed considerably lower sfGFP-MxCLP loading at a ratio of 2.5:1 than *in vivo* loading with 20 versus 31 copies of cargo, respectively (Figure 3B). In this experiment we were unable to analyze higher ratios due to low reassembling efficiencies and protein concentration.

### Scaffolding Effect in Mx_encap Reassembly

During initial *in vitro* sfGFP loading studies with different concentrations of sfGFP (with the corresponding CLP), it became apparent that cargo loading can promote Mx_encap *T* = 3 formation, while at high sfGFP concentrations, inhibition of capsid assembly was observed (Figure S3). To investigate this effect further, we repeated *in vitro* loading at a wider molar ratio range of sfGFP to Mx_encap monomer between 0 and 10:1 (Figure 2D). At molar ratios between 1:1 and 5:1, sfGFP-MxCLP addition promoted *T* = 3 reassembly over *T* = 1 (Figure 2D). At concentrations of above 5:1 molar ratio, capsids were exclusively observed as *T* = 3 size. However, reassembly efficiency started to be reduced above a 7.5:1 ratio (Figure 2D). It should be noted that the samples in these experiments have not been SEC purified before loading on BN-PAGE.

Particle measurement on TEM micrographs of SEC purified samples in Figure 3F–H gave further evidence for the *T* = 3 and *T* = 1 size distribution of Mx_encap. *In vivo*-assembled (unloaded) Mx_encap showed both *T* = 3 and *T* = 1 sizes as well as intermediate sizes that we were unable to separate with SEC (Figure 3H, denoted Mx_encap “purified”). These likely correspond to what can be seen as a discrete banding pattern between *T* = 1 and *T* = 3 (Figures 2C and S3 see *). Mx_encap reassembled without sfGFP (Figure 3H, denoted Mx_encap “in vitro noGFP”) showed both *T* = 3 and *T* = 1 sizes with the majority of the capsids in *T* = 1 symmetry. Upon addition of sfGFP-MxCLP at 2.5:1 molar ratio, a shift in diameter was observed toward the *T* = 3 symmetry (Figure 3H, denoted Mx_encap “in vitro 2.5:1”) while *in vivo*-loaded Mx_encap assembled completely into *T* = 3 capsids (Figure 3H, denoted Mx_encap “in vivo + GFP”).

Together our data suggest an apparent scaffolding effect, where cargo promotes Mx_encap assembly into *T* = 3 capsids *in vitro*. This scaffolding effect agrees with a recent *in vivo* study, observing two Mx_encap sizes in *E. coli* expression, with approximately 64% of particles in *T=* 3 and 36% in *T* = 1 symmetry.^23^ Upon coexpression of native cargo proteins, nearly 100% of capsids were in *T* = 3 symmetry. Another recent study^42^ showed that *in vivo* loading with exceedingly high concentrations of cargo resulted in the formation of distorted shells similar to our observations *in vitro* (Figure 3I).

### *In Vitro* Loading and Activity of Encapsulated Transketolase Homodimer

Critical to the industrial scope of using encapsulins as nanoreactors is an understanding of the stability of the loaded cargo. This is expected to be molecule specific, but general mechanisms such as molecular crowding, immobilization, and physical protection from degradation enzymes are conditions, which are expected to broadly enhance the stability of encapsulated cargo molecules. Here, we provide evidence for the encapsulation effect on the protein thermostability. Engineered sfGFP variants tend to be highly thermostable and are therefore not particularly suited to studying stability effects. We therefore chose a protein that is known to be less thermostable and whose stability can be measured as a function of activity, Transketolase enzyme TK (WP_061316063).

**Figure 4:**
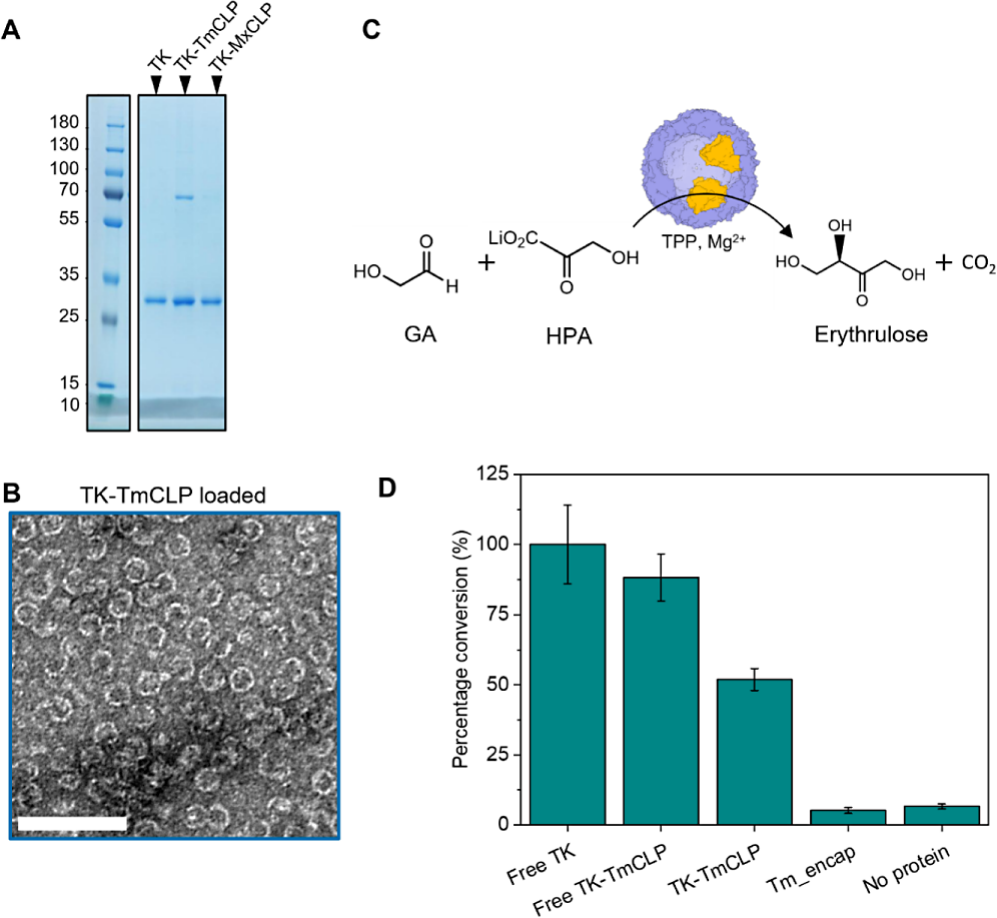
*In vitro* loading and enzyme activity of transketolase (TK) cargo. (A) Denaturing SDS-PAGE showing TK loading into *T. maritima* encapsulin. (B) TEM micrograph of TK-loaded *T. maritima* encapsulins at 1:1 molar ratio, from SEC fractions 8.5–14 mL (Figure S5) (scale bar 100 nm). Some incomplete capsids can be seen. (C) Erythrulose reaction with TK (yellow) loaded into Tm_encap, TK cofactor thiamine pyrophosphate (TPP) and Mg^2+^. TK activity was measured as the formation of erythrulose from the 3-carbon ketol donor β-hydroxypyruvate (HPA) and 2-carbon acceptor glycolaldehyde (GA). (D) Transketolase activity comparing free TK (100%), free TK-TmCLP and encapsulated TK-TmCLP in Tm encapsulin (TK-TmCLP). Column plots show the mean values with error bars from standard deviations based on three replicates.

Due to the low efficiency of *in vitro* Mx_encap reassembly, we proceeded with Tm_encap, which was loaded with TK via the C-terminal Tm cargo loading peptide at a 1:1 ratio, and residual TK was subsequently removed via SEC purification. Similar to sfGFP, apo-transketolase loading was only possible with the correct TmCLP, and no passive loading or cross loading was visually observed (Figure 4A). TEM analysis of TK-TmCLP-loaded Tm_encap revealed the presence of both closed and open compartments (Figure 4B), resembling the disrupted structures observed with sfGFP loading (Figure 3C). It is possible that the TK cargo destabilizes the capsid, potentially rendering the sample more fragile and susceptible to damage during TEM sample preparation. The Tm_encap loaded with TK exhibited a comparable SEC profile and hydrodynamic radius to assembled Tm_encap (Figures S5 and S6), suggesting minimal impact on its overall structural integrity. Due to the required sample concentration for the experiment, we opted to collect the broad SEC peak (8.5–14 mL), which presumably also encompasses open structures as the SEC column does not achieve complete resolution of the Tm_encap peak from potential incomplete products.

The TK is a homodimer with two identical active sites formed at the homodimer interface. TK enzymatic activity was measured to confirm that TK remained in its active conformation with the C-terminal fusion of the *T. maritima* CLP and following loading into the capsid. The end point activity of TK was measured as the formation of erythrulose from the 3-carbon ketol donor β-hydroxypyruvate (HPA) and the 2-carbon acceptor glycolaldehyde (GA) (Figure 4C). The required TK cofactors, thiamine pyrophosphate (TPP) and MgCl_2_, were excluded during loading and capsid reassembly due to absorbance at 280 nm and interference with subsequent SEC. Instead, they were incubated with samples at saturation concentrations 30 min prior to the addition of the substrates. The addition of the *T. maritima* CLP was not found to significantly affect the activity, showing 88% activity compared to the free TK (Figure 4D). Encapsulation in Tm_encap retained TK activity, demonstrating that the loaded TK was in its functional dimeric holoenzyme form and not inactivated during reassembly; however, a 50% drop in end-point activity was seen (Figure 4D). This is likely the result of diffusion limitation for substrate and cofactors across the encapsulin compartment as noted by previous literature. The pore of Tm_encap has a diameter of 3 Å, which acts as a significant electrostatic diffusion barrier to metal ions.^17,38,43^ However, the loops are intrinsically flexible and have been shown in the *Haliangium ochraceumT* = 1 encapsulin to have an open conformation of 15 Å.^44^ Modifications can be made to effectively enlarge the pore diameter to facilitate efficient transport of metabolites, which has been demonstrated by deletions from the pore forming loop in the capsid protein from *T. maritima*,^43,45^ and which led to a notable increase in pore diameter to 11.3 Å (S7G mutant, PDB: 7LIT).^45^ It was demonstrated that the combination of pore size and charge parameters is responsible for regulating the flow though pores. One potential avenue is the systematic design of the nanoreactor porosity, which could be adjusted for a variety of chemicals.

### Investigating the Stability of *In Vitro*-Loaded Transketolase via Conjugated FRET Fluorophores

**Figure 5:**
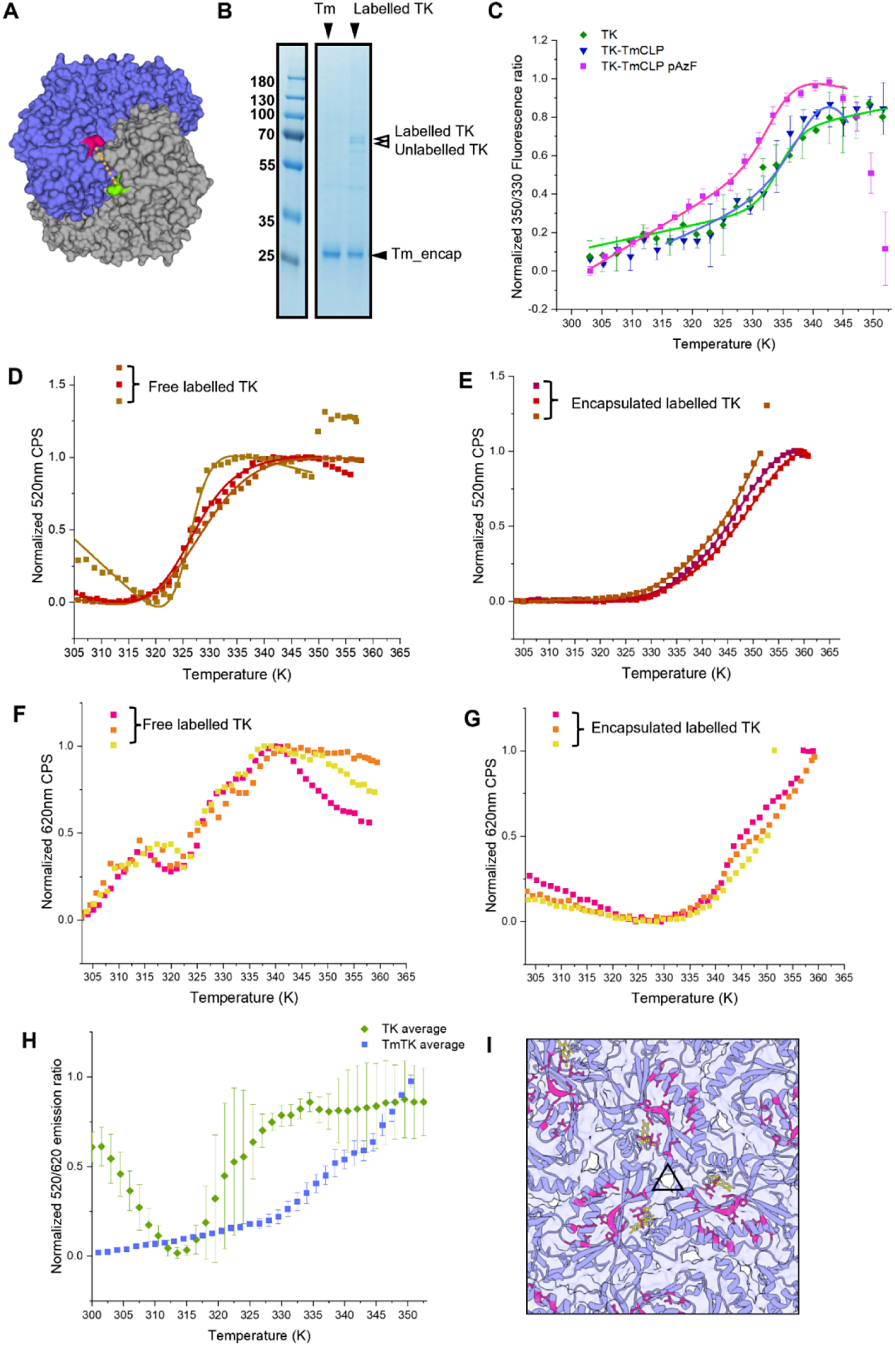
FRET assay of *in vitro*-loaded transketolase. (A) TK homodimer with K603pAzF-labeled position shown in green and magenta, illustrating the FRET donor AF488 and acceptor AF594, respectively. The α carbon distance indicated at 19 Å. (B) SDS-PAGE of Tm-encapsulin only and encapsulated FRET dye-labeled TK-TmCLP K603pAzF. White arrows highlighting two bands associated with labeled and unlabeled TK-TmCLP K603pAzF. (C) Normalized 350/330 nm ITF ratio of transketolase (TK), TK fused with TmCLP (TK-TmCLP), and TK-TmCLP with NcAA incorporation (TK-TmCLP K603pAzF). Thermal ramp rate at 4 °C per minute. (D, E) Normalized FRET donor data at 520 nm emission of free (D) and encapsulated (E) labeled TK-TmCLP K603pAzF. Normalized plots fitted Van‘t Hoff two-state transition. Different colored data points indicate individual repeats. (F,G) Normalized FRET acceptor data at 620 nm emission of free (F) and encapsulated (G) labeled TK-TmCLP K603pAzF. 620 nm emission showed a multistate transition and could not be fitted. Different colored data points indicate individual repeats. (H) FRET emission ratio 520/620 of free and encapsulated labeled TK-TmCLP K603pAzF with the same thermal ramp rate. (I) Luminal view on the *T. maritima* encapsulin at the three-symmetry axis. Bound FMN is shown in yellow, and residues involved in cargo binding are highlighted in magenta.

To determine the stability gain through *in vitro* loading of TK, we used a FRET pair at positions within the protein shown to correlate with global stability of TK homodimer.^46^ The FRET signal probes the local relative conformational changes between the two C-terminal domain residues at position 603, across the dimer interface (Figure 5A). FRET analysis enabled us to deconvolute TK’s thermal transition midpoint from the surrounding compartment shell protein responses.

FRET labeling required noncanonical amino Acid (NcAA) incorporation. We carried out site directed mutagenesis of the TK K603 position to the TAG (Amber) stop codon to allow for incorporation of *para*-azidophenylalanine (pAzF) when coexpressed with the appropriate refactored tRNA aminoacyl synthase machinery. Expression tests were carried out in BL21 Star (DE3) and the “Amberless” C321 *E. coli* strains.

BL21 Star (DE3) expression yielded only truncated, insoluble TK-TmCLP (Figure S7A). A proportion of full length and truncated TK-TmCLP was expected due to competition between TAG cognate release factor 1 (RF1) and refactored NcAA pAzF tRNA. However, no soluble full-length protein was produced. While cell mass and total protein yield were lower in the C321 strain, and a proportion of truncated TK-TmCLP was seen despite the RF1 deletion, C321 expression yielded full length TK-TmCLP, indicating incorporation of pAzF at the 603 position (TK-TmCLP K603pAzF) (Figure S7A). The full-length protein was IMAC-purified and concentrated (Figure S7B).

Next, TK-TmCLP K603pAzF was labeled with Alexa Fluor dyes AF488 and AF594, which resulted in a >85% loss in protein yield to the membrane during the dialysis phase. As a result, the molar ratio used during *in vitro* loading was limited to 1:1 of dye-labeled TK-TmCLP K603pAzF per encapsulin monomer. Following SEC purification of loaded encapsulins, two SDS-PAGE bands were visible, which is consistent with dye-labeled and unlabeled subpopulations of TK-TmCLP K603pAzF with a molecular weight shift of +0.77 kDa and +1.23 kDa for AF488 and AF594, respectively (Figure 5B).

### Increased Thermal Stability of Encapsulated TK

The 350/330 nm fluorescence emission ratio from intrinsic tryptophan fluorescence (ITF) thermal-denaturation measurements on free TK, TK-TmCLP, and TK-TmCLP K603pAzF indicated that the TmCLP addition had no effect on the global stability of the TK homodimer, with *T*_m_ values of 334 K (60.85 °C). The NcAA incorporation had a minimal destabilizing effect with a *T*_m_ of 333.8 K (60.65 °C), consistent with previous observations when labeled at this site.^46^ The FRET AF488 and AF594 systems have an emission maximum of 520 nm for the FRET donor AF488 and 620 nm for the acceptor AF594. Within a maximum distance, excitation at 488 nm will be resonantly transferred from the donor to acceptor, resulting in a 620 nm emission and quenching of the 520 nm emission. As the system decouples, the 520 nm emission should unquench, and a corresponding drop in 620 nm emission is expected.

In both the free and encapsulated states, dye-labeled TK-TmCLP K603pAzF displayed a 520 nm transition, with free TK displaying a cooperative transition at 330 K and the encapsulated TK showing a broad uncooperative transition with a significantly increased *T*_m_ value at 345 K (Figures 5D,E and S8A,B).

The free TK showed a three-state transition of the 620 nm FRET acceptor signal, indicating a new intermediate state at approximately 315–320 K (Figures 5F and S8C). This additional transition was detected by the acceptor-probe emission at 620 nm (Figure 5F), indicating a structural event local to the acceptor probe. The main transition still occurred between 325 and 330 K, consistent with the two-state transition measured at 520 nm (Figure 5D).

For the encapsulated TK, however, the transition at 620 nm remained quasi-two-state (Figures 5G and S8D) but without an unfolded signal end point, and so the *T*_m_ was estimated at >340 K, consistent with the 520 nm transition (Figure 5E). The difference in 620 nm donor emission profiles between the free and encapsulated TK (Figure 5F and G) indicates an increase in cooperativity of the 620 acceptor and, by implication, a suppression in conformational freedom with respect to free TK.

To determine whether the increases in 620 nm emission observed were simply spectral overlap with the 520 nm probe emission absorbance, the 520/620 ratio was plotted (Figure 5H). If driven by FRET donor 520 nm emission, we would expect a proportional relationship, otherwise evidence of a transition would indicate a change in electronic coupling. For both the encapsulated and free TK, we saw distinct transitions. For free TK, the 520/620 nm profile showed a steep initial baseline and then a denaturation transition *T*_m_ at 320.5 ± 2.3 K. When encapsulated, a distinct local 520/620 nm transition is seen within the overall upward trend, distinctly higher than the local free TK 520/620 nm signal with a *T*_m_ of 335.2 ± 2.7 K (Figure 5H). This corroborates the positively shifted *T*_m_ observed between the free and encapsulated TK at 520 nm (Figure 5D,E).

Tm_encap is a flavin-binding protein with the FMN-binding cleft located near the trimeric order of symmetry, coordinated primarily through tryptophan residue 90 (Figure 5I). The FMN electronic structure complicates the FRET pair system, as the oxidized free excitation spectra may overlap the FRET donor AF488 absorbance maxima at 350 and 450 nm and the fluorescence maxima at 530 nm. It was reported that the fluorescence of Tm_encap-bound FMN is strongly quenched, likely through π-stacking by the aromatic protein binding cleft^47^ and indeed was not seen in the excitation and emission scans of 0.5 mg/mL Tm_encap (Figure S9). Furthermore, despite the strong yellow color of purified Tm_encap, once reassembled at comparable concentrations, the intensity of yellow color is reduced. The predicted interference of the bound FMN would therefore be reduced after disassembly and reassembly, and while coordinated, the interference is likely to be limited to coabsorb at 488 nm, acting as an additional quenching interaction of the FRET donor and not effecting the development of the 620 nm emission signal.

The reassembled encapsulin control (Figure S8B) showed no significant increase in 520 nm emission up to 80 °C, in line with its well-known characteristics as a heat stable protein. While FMN interference cannot be ruled out, the difference in signal profile likely reflects the extreme difference in protein environment, in which TK becomes highly confined against unfolding due to encapsulation. Once encapsulated, the TK homodimer is confined to an internal luminal diameter of the Tm_encap of <20 nm. One TK homodimer has a diameter of approximately 10.3 nm^48^ and so is consistent with the 2.6 dimers per capsid on average. Such confinement of 2 or 3 TK homodimers may lead to additional 620 nm emission due to FRET from adjacent colocalized dimer fluorophore coupling, which is likely to have additional transitions as TK dimers denature within the confined internal volume.

## Discussion

Using direct observation of capsid reassembly via BN-PAGE, we have seen that Tm_encap and Mx_encap show reversible assembly under different denaturant conditions. Tm_encap showed a complete disassembly in low and high pH conditions and partial disassembly in 6 M GuHCl and was resistant to 8 M urea. Mx_encap readily disassembled in all conditions apart from acidic pH treatment, which caused insoluble precipitation, but only displayed observable reassembly after disassembly in 8 M urea. This stark difference in denaturant conditions was not expected, given the shared fold and sequence similarity. The pH tolerance may reflect the native extremophilic conditions of *T. maritima*. GuHCl and urea have mechanisms similar to those of chaotropic agents, but GuHCl is a stronger denaturant with a higher ionic strength. Interestingly, Tm_encap denatured samples showed an BN-PAGE band slightly above the 66 kDa native marker (Figure 1B), indicating potential dimers (∼65 kDa). This may indicate that incubation in NaOH does not fully disassemble *T. maritima* encapsulins into monomers but rather to dimers. Dimers have been proposed as the first complex of the *B. linens* encapsulin assembly pathway, which shares a rotated E loop structure with Tm_encap whose domain swaps between capsomers, possible due to the loop rotation.^13^ Interestingly the Mx_encap *T* = 1 shares a rotated E loop structure,^23^ but similar dimer bands were not seen in BN PAGE gel. In terms of reassembly efficiency, Tm_encap showed high recovery of compartments, following a simple 0.15 M NaOH treatment, without the need for expensive excipients or dialysis steps possibly allowing facile scaling of the process. Mx_encap showed poor efficiency under the conditions tested. Only with the addition of targeted cargo was the reassembly improved.

*In vitro* loading of Mx_encap exhibited a dual relationship to cargo concentration: a scaffolding effect at low concentrations promoting *T* = 3 symmetry and an inhibition of reassembly at high concentrations of cargo protein. This observed scaffolding effect of cargo is likely similar to HK97 bacteriophage major capsid protein scaffolding mechanisms, which prevent premature closure of the capsid allowing larger conformations to assemble.^49−51^ The binding cleft in the Mx_encap has recently been determined near the 2-fold symmetry interface with primarily intercapsomer interactions. Additionally, ionic interactions between the native CLP and neighboring shell monomers add further evidence for a cargo-mediated scaffolding role.^42^

Tm_encap and Mx_encap *in vitro* loading demonstrated cargo selectivity, with no sfGFP loading observed and little cross-loading of sfGFP with a non-native CLP tag. While this is not surprising given the differences in the CLP sequence and monomer cleft,^52^ the efficient exclusion of incorrect loading at high contaminant concentrations opens the possibility to selective loading in mixed reassembly systems.

Tm_encap loading stoichiometry increased with increasing cargo concentration during reassembly with maximum cargo band intensity at a 15 × molar ratio with 15 molecules loaded. Compared to the *in vivo*-loaded control at 2.94 copies, this is a 5-fold increase in number of molecules loaded. The assumption is that *in vivo* loading would have a more appropriate loading, with higher local concentrations of sfGFP and chaperone proteins around the translational machinery and assembling encapsulins. For native cargos, Cryo-EM density studies of ferritin like protein (FLP) encapsulins from *Haliangium ochraceum* and *T. maritima* have shown up to 4 or 5 FLP decamers.^44,47^ However, for fluorescence reporter cargos, low loading stoichiometries have been reported both *in vivo* and *in vitro* in *T* = 1 compartments likely due to the difference in oligomerization state of the cargos.^15,31,32,38^ Taken together, our observation of increasing loading at higher concentrations may indicate that *in vitro* loading is driven by concentration of cargo but also limitation of available monomers as both Mx_encap and Tm_encap showed a decrease in capsids at higher cargo concentration. To achieve high non-native cargo loading titers, appropriate cargo ratio during reassembly is required. The reason for *in vitro* loading exceeding *in vivo* loading is not known; however, possible explanations could be that larger compartments are formed in the presence of excessive cargo or that the compartments do not achieve closure. In both these scenarios, a greater amount of CLP binding clefts would be exposed and not sterically blocked. This first scenario has been observed recently *in vivo.* Cryo-EM of *in vivo* high loading Mx_encap constructs was observed to break *T* = 3 symmetry and form frustrated larger *T* = 3 assemblies compared to low loading constructs.^42^ In this study, we have observed the latter case, of visually nonclosed structures via TEM under *in vitro* high cargo molar ratios (Figure 3D). Cargo-mediated frustration of the shell compartment has previously been reported where cargo loading was shown to reduce mechanical stability of the compartments, which may slow closure of the capsid.^13^ Interestingly, a 1:1 ratio of cargo to monomer, with the expression of AMP-encapsulin fusion protein was shown to prevent compartment closure but resulted in discrete soluble structures.^53^ A methodology to determine encapsulin closure “fitness” would be interesting for *in vitro*-loaded capsids, such as a small protease challenge or native mass spectroscopy. Recently reassembled encapsulin’s resistance to rupture was measured via AFM, which showed significant decrease in pressure post reassembly, perhaps indicating a significant population of high assembly order complexes.^12^ Ultimately, the data provided here are only a relative measure as cargo loading is likely to be highly heterogeneous across capsids. Techniques such as native mass spectrometry and cryo-EM could provide a more detailed characterization of the uniform stoichiometry.

We have attempted to probe the effect that encapsulation has on relevant cargo with regards to thermal stabilization. Using orthogonal FRET-labeled TK, we used the K603 local interface stability as a proxy for the global TK homodimer stability. It is important to show that the local stability transition aligns with the global transition determined via the ITF to justify its use as a proxy. The TK FRET positions seem to behave differently from what was previously observed, with the 620 nm emission having a multistate transition with an overall upward trend. This may indicate low labeling affinity of the AF594 acceptor compared to the AF488 donor with the upward trend driven by residual AF488 signal or otherwise aggregation behavior rather than cooperative two-state thermal melting. To rule out residual 520 nm emission, the 520/620 nm emission ratio was plotted, which should show a consistent trend if 620 nm emission was driven by the AF488 donor. The 520/620 nm profile indicates that the observed upward trend of 620 nm acceptor emission is not entirely driven by residual donor emission, with a distinct transition. Regardless, the 620 nm signal is acting as a direct reporter on the local changes to the relative positions of the 603 sites on the TK homodimer. The free TK FRET transition is significantly lower than the global transitions measured via ITF. When encapsulated, a distinct local 520/620 nm transition significantly higher than that of the local free TK is seen within an overall upward trend, with a *T*_m_ similar to a global *T*_m_ temperature seen at approximately 335 K. While this transition shift likely reflects a stabilizing effect of being encapsulated, the difference in protein environment makes direct comparison difficult. Furthermore, Tm_encap is a FMN binding protein, which may complicate the FRET system of the encapsulated encapsulin with FMN Ex/Em profile overlapping the FRET donor. It is known that while bound by the encapsulin, the fluorescence is strongly quenched by aromatic residues involved in the binding cleft, which we observed by the 530 nm emission maxima not present in the 3D scan of the Tm_encap. While bound, the influence therefore should be limited to additional parasitic absorbance at 488 nm shown by the control of unloaded *T. maritima*, showing negligible increase in 520 nm emission up to 80 °C, indicating no significant FMN release, in line with its well-known characteristic as a heat stable protein.

While FMN interference cannot be ruled out, the difference in signal profile probably reflects the extreme difference in protein environment or’compactness’ of encapsulation. Taken together, these data suggest that there is a significant thermal stabilizing effect for encapsulated TK within encapsulin capsid with the *T*_m_ shifted +15 °C from the FRET profile. However, given the difference in protein environment, how well the 603 position relates as a proxy for global stability once encapsulated may be significantly different. While the nature of the delayed transition is not fully known, a stabilizing effect due to the local high concentration due to encapsulation is highly likely.

## Conclusion

To our knowledge, encapsulin physiochemical stability studies have been focused on the capsid protein and not the cargo. In this study, we have developed consistent *in vitro* loading protocols that allowed us to apply an orthogonal NcAA FRET stability assay method to deconvolute the loaded cargo stability transitions from the surrounding capsid proteins. From the results, we concluded that the constraint environment within the capsid has an impact on TK stability increasing loaded TK thermal midpoint by +15 °C in a noncooperative manner. The use of similar FRET systems could be further applied to other nonprotein biomolecules cargo in the future.

## Material and Methods

### Cloning of Encapsulin Constructs

The capsid protein sequences used in this study were derived from *Thermotoga maritima* and *Myxococcus xanthus* encapsulins. The *Thermotoga maritima* encapsulin sequence is derived from the iGEM BioBrick registry (BBa_K2686001). The encapsulin gene-containing plasmid used it this study had been generated in previous work.^28^ The *Myxococcus xanthus* encapsulin sequence was taken from the *Myxococcus xanthus* genome (MXAN_3556).^54^ The gene was ordered as a gBlock from Integrated DNA Technologies (IDT) and subcloned into the pET3a plasmid under a T7 promotor (Novagen). Both encapsulin proteins contain a C-terminal fusion composed of a flexible GSG linker followed by the 8 residue Strep-Tag II and were codon-optimized for expression in *Escherichia coli* (Tables S1 and Table S2, sequences in Supporting Information).

### Cloning of Superfolder GFP and Transketolase Cargo

The superfolder GFP (sfGFP) sequence was ordered from Addgene in the pJL1 expression vector under a T7 promotor (Addgene:69496). A hexa-histidine tag was fused onto the N terminus of sfGFP, and the encapsulin-specific minimal cargo loading peptides (CLPs) were fused onto the C terminus of sfGFP, both by overhang PCR. The following CLPs were used: *T. maritima* CLP – GGSENTGGDLGIRKL^15^ and *M. xanthus* CLP – LTVGSLRR.^18^ Gibson assembly was used to generate the pJL1-sfGFP_TmCLP-Tmencap_STII and pJL1-sfGFP_MxCLP-Mx_encap_STII plasmids for coexpression and *in vivo* loading of sfGFP into Tm_encap and Mx_encap, respectively (Tables S1 and Table S2, sequences in Supporting Information).

The *E. coli* transketolase sequence (WP000098614.1)^54^ was fused with the *T. maritima* CLP at the C terminus via PCR using the primers listed in Table S1 and following New England Biolabs (NEB) KLD (Kinase, Ligase, DpnI) protocol. TK_TmCLP was subcloned into the pQR1622 plasmid, via NheI and XhoI sites, under a T5 (sigma70) constitutive promoter, and the final construct was named pQR1623. pQR1622 was constructed and gifted by J. Ward at the Department of Biochemical Engineering, UCL.

### Site Directed Mutagenesis for Noncanonical Amino Acid (NcAA) Incorporation

His-Transketolase-TmCLP was mutated at lysine 603 to a TAG amber stop codon (K603TAG), using the NEB Q5 Site-Directed Mutagenesis Kit (Table S1).

### Encapsulin Expression and Purification

Encapsulin gene containing plasmids were transformed into chemically competent BL21 Star (DE3) *E. coli* cells (ThermoFisher Scientific). 10 mL of starter cultures was prepared from a single colony in Luria–Bertani (LB) medium with appropriate antibiotic at standard working concentrations, at 37 °C degrees for 16 h overnight. Starter cultures were inoculated into 500 mL of LB at a starting OD_600_ of 0.05 and cultured at 37 °C at 180 rpm shaking. Protein expression was induced for 18 h overnight with 400 μM isopropyl β-d-1-thiogalactopyranoside (IPTG) (ThermoFisher Scientific). Cells were harvested by centrifugation at 6,000*g* for 10 min and resuspended in 25 mL of buffer W (100 mM Tris-Cl pH 8.0, 150 mM NaCl, 1 mM EDTA). Cells were lysed on ice by sonication (15 cycles, 10 s on, 10 s recovery). Cell debris was removed via centrifugation at 18,000*g* at 4 °C, and the soluble fraction was affinity-purified via 5 mL Strep-TactinXT 4Flow (IBA) gravity column. Encapsulins were eluted in 3 CV BXT buffer (0.1 M Tris-Cl pH 8.0, 0.15 M NaCl, 50 mM Biotin, 1 mM EDTA) and stored at 4 °C. Typically, a 500 mL culture yielded approximately 1–3 mg of encapsulins after pooling all fractions from the 5 mL Strep-Tactin column. Hexa-histidine-tagged sfGFP was purified from 250 mL cultures via immobilized metal affinity chromatography (IMAC) using 5 mL nickel-charged Chelating FastFlow Sepharose resin gravity columns (GE Healthcare). Wash steps followed a stepwise 10–100 mM imidazole gradient in 20 mM Tris-Cl pH 7.5, 0.5 M NaCl, with final elution in 2 CV 500 mM imidazole. Eluted fractions were confirmed via SDS-PAGE and buffer exchanged via ammonium sulfate precipitation at a final concentration of 3.9 M. The precipitated protein was stored at 4 °C.

### Transketolase Expression and Purification

Transketolase plasmid pQR1623 was transformed into chemically competent DH5α cells and expressed from a single colony in a 10 mL Luria–Bertani (LB) overnight starter culture with appropriate antibiotic at 37 °C, before inoculating 500 mL of cultures at an OD_600_ 0.05 and grown at 37 °C and 180 rpm shaking for 18 h. Lysis and IMAC purification were performed as described above.

### Transketolase K603(TAG) *Para*-Azido Phenylalanine (pAzF) Noncanonical Amino Acid (NcAA) Incorporation

The pQR1623_HH_TK-K603(TAG)-TmCLP plasmid was cotransformed with the pULTRA-CNF plasmid containing the aminoacyl tRNA synthetase gene and tRNA gene into chemically competent C321 *ΔA exp* “Amberless” *E. coli* strain with all genomic UAG Amber codons replaced to UAA and the Amber associated release factor (RF1) gene deleted. C321 was cultured from a single colony in 5 mL of Terrific Broth (TB) overnight starter culture with appropriate antibiotic at 37 °C, before inoculating 250 mL of TB cultures at an OD_600_ of 0.05. The growth media were supplemented with 1 mM pAzF (pAzF 100 mM stock dissolved in 0.5 M NaOH) and 1 mM IPTG for strong expression of aminoacyl tRNA synthetase (aaRS) under the lactose inducible tac1 promotor to produce tRNA for pAzF incorporation. The cultures were grown for 18 h at 37 °C and 180 rpm, harvested and lysed as described above. Ammonium sulfate precipitation was not performed post IMAC purification due to yield losses.

### Disassembly, Reassembly, and *In Vitro* Loading

For disassembly, *T. maritima* and *M. xanthus* encapsulins were precipitated with final concentration of 10% PEG 8,000 and 0.5 M NaCl for 1 h on ice and centrifuged at 18,000*g* for 30 min. The supernatant was aspirated off, and the encapsulin pellet was gently resuspended. Tm_encap was resuspended in 150 mM NaOH, and *M. xanthus* was resuspended with freshly prepared 8 M urea. The volume was adjusted to a final monomer concentration of 100 μM, and the samples were incubated for 1 h on ice.

Reassembly of encapsulin proteins was initiated by 10 times dilution of denaturant conditions with reassembly buffer (0.3 M Tris-Cl pH 7.5, 0.15 M NaCl) with 5 mM beta-mercaptoethanol (BME). Reassembly reactions were incubated at 4 °C overnight with a final monomer concentration of 10 μM. sfGFP and transketolase *in vitro*-loaded variants were precipitated with ammonium sulfate and then buffer-exchanged into 0.3 M Tris-Cl pH 7.5, 0.15 M NaCl. Residual ammonium sulfate was removed from transketolase samples via PD-10 desalting columns equilibrated with the reassembly buffer. Cargo proteins were concentrated using Viva spin 20, 10,000 MWCO PES membrane columns (Sartorius) and centrifuged in a swing bucket rotor at 4,000*g*. Dilutions of sfGFP and transketolase were prepared in reassembly buffer with 5 mM BME, and denatured encapsulins were added and allowed to reassemble overnight at 4 °C. Post reassembly, Blue Native (BN)-PAGE and size exclusion chromatography (SEC) were performed to verify loading and to remove the unloaded cargo protein.

### Blue Native-PAGE (BN-PAGE)

BN PAGE of reassembled encapsulins was performed using Native-PAGE 3 to 12% Bis-Tris gels (ThermoFisher Scientific) with light blue cathode buffer (0.002% G-250 Coomassie) on ice at 150 V for 120 min. Prior to loading, samples were mixed and diluted with 4 × sample buffer (50 mM BisTris, 50 mM NaCl, 10% w/v glycerol, 0.001% Ponceau S, pH 7.2). Gels were quickly washed with water before fluorescence imaging using the Amersham imager 600RGB (GE Healthcare) CY2 blue channel in semi-Auto mode. Excessive fluorescence from unloaded sfGFP variants was masked with aluminum foil. Subsequent Coomassie staining used InstantBlue (Expedeon) gel stain.

### Size Exclusion Chromatography (SEC)

Reassembled encapsulins were polished using a Superose 6 10/300 GL (Cytiva) column on the ¨KTA Pure chromatography system (GE Healthcare) with a running buffer of 20 mM Tris-CL, 50 mM NaCl, pH 7.5. 2 mL of fractions was collected, and fractions containing encapsulin bands were pooled and concentrated via Viva 20 10,000 MWCO PES Spin columns (ThermoFisher Scientific).

### SDS-PAGE Densitometry

SDS-PAGE densitometry was used to quantify the molar ratio of coeluted sfGFP or transketolase cargo with reassembled encapsulins. Standard curves were generated by loading known concentrations (0.01–1 μg) of purified encapsulins and either sfGFP or transketolase and band intensity plotted. *In vitro*-loaded samples were loaded at 1 μg/lane and plotted against the standard curves. All gels were Novex WedgeWell 12% Tris-glycine protein gels (Invitrogen) and stained using InstantBlue (Expedeon) gel stain.

### Transketolase Activity Assay

Transketolase activity assay measured the irreversible development of erythrulose from glycolaldehyde (GA), with β-hydroxypyruvate (HPA) as the ketol donor. 50 μL of purified transketolase at 0.06 mg/mL was incubated for 30 min, with cofactors at saturation concentration of 100 μL of 3.6 mM thiamine pyrophosphate (TPP), 14.7 mM MgCl_2_, 50 mM Tris-Cl, pH 7.5. The reaction was initiated with the addition of 150 μL lithium β-hydroxypyruvate and glycolaldehyde at 200 mM dissolved in 50 mM Tris-Cl, pH 7.5. All reactions were performed in triplicate and incubated for 1 h at 22 °C with agitation at 300 rpm before quenching 50 μL in 450 μL 0.1% v/v TFA. Samples were centrifuged at 10,000*g* and the supernatant analyzed via HPLC with Bio-Rad Aminex HPX-87H normal phase column (Bio-Rad) at 0.6 mL/min of 0.1% TFA mobile phase. Absorbance was monitored at 210 nm, and peak areas were analyzed via ThermoFisher Scientific Dionex Chromeleon 7.2 software.

### Negative Stain TEM

3 μL of assembled/reassembled encapsulin samples were aspirated on to Formvar Carbon 400 Mesh Copper grids (Agar scientific) and allowed to dry under a lamp for 3 min. Excess liquid was removed, and the grid was washed by transfer onto a RO water droplet, blotted, and transferred on to a filtered 1% uranyl acetate droplet for 30 s before excess stain was removed and the grid was allowed to air-dry. Stained grids were imaged on a Joel JEM-1010 transmission electron microscope. The converted TIFF files were analyzed via FIJI ImageJ v.1.53c software distribution (National Institutes of Health, USA).

### Transketolase Labeling with DIBO Alkyne AlexaFluor 488 (AF488) and AlexaFluor 594 (AF594)

IMAC-purified and buffer-exchanged transketolase-TmCLP K603pAzF was labeled at a 7-fold molar excess with both AF488 and AF594 DIBO alkyne dissolved in DMSO, in a single reaction protected from light overnight at room temperature with a final DMSO concentration of 8.5%. Labeled protein was dialyzed from free AF labels by four dead volume spins in Amicon Ultra-4, regenerated cellulose 10,000 MWCO membrane (Millipore) and buffer-exchanged into the reassembly buffer. This step unfortunately resulted in an 88% loss in protein based on protein concentration before labeling. The resulting labeled protein concentration was quantified via Nanodrop absorbance at 280 nm corrected for the 280 nm absorbance of the AF labels.
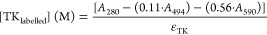


0.11 and 0.56 are correction factors at 280 nm absorption of AF488 and AF594, respectively. ∑_TK_ is the extinction coefficient for TK_labeled_.

### Intrinsic Tryptophan Fluorescence Measurements

The melting temperature (*T*_m_) values of the Transketolase variants were determined via intrinsic tryptophan fluorescence (ITF) emission ratio 350/330 nm, with a linear temperature ramp at either 1 or 4 °C/min between 30 and 90 °C. ITF measurements used the UNit and UNcle (Unchained Laboratories, UK) protein stability system using UNi microcuvette arrays. Excitation was achieved by a 266 nm laser without plate hold. 350/330 ratio thermal melt data were fitted to a two-state Van’t Hoff model^55^

### Thermal Denaturation Measurements of Transketolase via the AlexaFluor 488–594 FRET System

FRET emissions were measured using the FluoroMax4 (HORIBA) spectrofluorometer with a SC150 immersion circulator fitted to a temperature regulated cuvette holder. An average linear 4 °C/min thermal ramp rate between 30 and 80 °C was determined by measuring the rate of temperature change in triplicate of two thermocouple probes in a water cell. This was directly attached to the cuvette holder, while heating between 20 and 95 °C SC150 immersion water bath set points. The Thermocouple probes were measured every 20 s using Pico Technology TC-08 thermocouple data logger, and the average linear correction factor between cuvette holder temperature and water sample temperature was determined. Point emission measurements at 520 and 620 nm were collected every 20 s over the temperature ramp. The excitation and emission slit width settings and the JY batch experiment loop time delay were variable on the initial measured fluorescence photon count rate (CPS). Free-labeled transketolase was diluted to 0.5, 0.1, and 0.01 mg/mL, and all transketolase samples were incubated with cofactors at saturation concentrations. 10 μL of protein sample was loaded into a 1 mm × 1 mm quartz cuvette and layered with silicon oil to prevent evaporation. Samples had a 3 min equilibration time and measured in triplicate. The 520 nm emission data was normalized and individually fitted to a two-state Van’t Hoff model.^55^

## Supporting Information Available

Tm_encap purification (Figure S1), sfGFP cargo loading and scaffolding effect (Figures S2, S3), SEC profiles of sfGFP *in vitro* loading into Tm_encap and Mx_encap (Figure S4), SEC profiles of transketolase *in vitro* loading (Figure S5), DLS size frequency distributions by volume of reassembled Tm_encap with and without TK (Figure S6), expression studies for pAzF incorporation (Figure S7), raw FRET donor and acceptor signal development data (Figure S8), excitation and emission 3D scan of Tm encapsulins (Figure S9), primer (Table S1), plasmid and strain information (Table S2), and DNA and amino acid sequences of genes used in study (PDF)

## Abbreviations

CLP: cargo loading peptide
FLP: ferritin like proteins
FMN: flavin mononucleotide
FRET: Förster resonance energy transfer
GFP: green fluorescent protein
GuHCl: guanidium hydrochloride
ITF: intrinsic tryptophan fluorescence
Mx_encap: *Myxococcus xanthus* encapsulin
MxCLP: *Myxococcus xanthus* specific cargo loading peptide
NcAA: noncanonical amino acid
pAzF: *para*-azidophenylalanine
sfGFP: superfolder green fluorescent protein
TEM: transmission electron microscopy
TK: transketolase
*T*_m_: protein thermal melt transition midpoint
Tm_encap: *Thermotoga maritima* encapsulin
TmCLP: *Thermotoga maritima* specific cargo loading peptide
