# Supplementary material for: Encapsulation of Transketolase into In Vitro-Assembled Protein Nanocompartments Improves Thermal Stability
Source: ACS Appl Bio Mater. 2024 Jun 5;7(6):3660–74. doi: 10.1021/acsabm.3c01153 (PMC11190991; doi:10.1021/acsabm.3c01153)
Supplement: Supplementary file 1 — mt3c01153_si_001.pdf [file mt3c01153_si_001.pdf]

SUPPORTING INFORMATION

Encapsulation of transketolase into *in vitro*-assembled protein nanocompartments improves thermal stability

Alexander Van de Steen[1†], *Henry C. Wilkinson*[1†], *Paul A. Dalby*[1], *Stefanie Frank*[1*]

[1]University College London, Department of Biochemical Engineering, Bernard Katz Building, Gower Street, WC1E 6BT, London, UK

*Correspondence: stefanie.frank@ucl.ac.uk

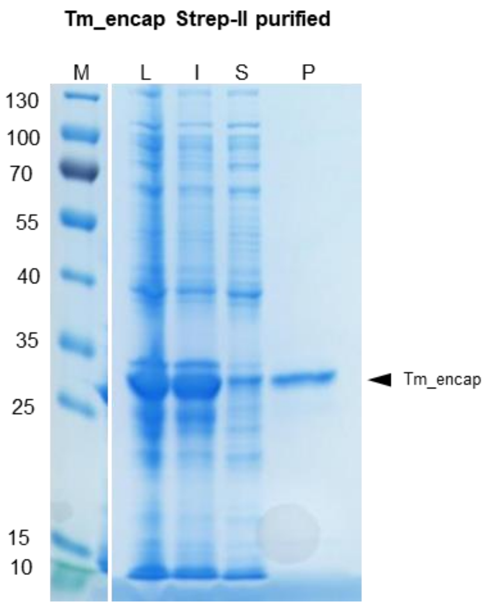

Figure S1: Strep-Tactin®XT purification of Tm_encap. M=molecular weight marker, L=cell lysate, I=insoluble fraction after clarification of lysate, S=soluble fraction clarification of lysate, loaded on Strep-Tactin®XT column, P=purified fraction.

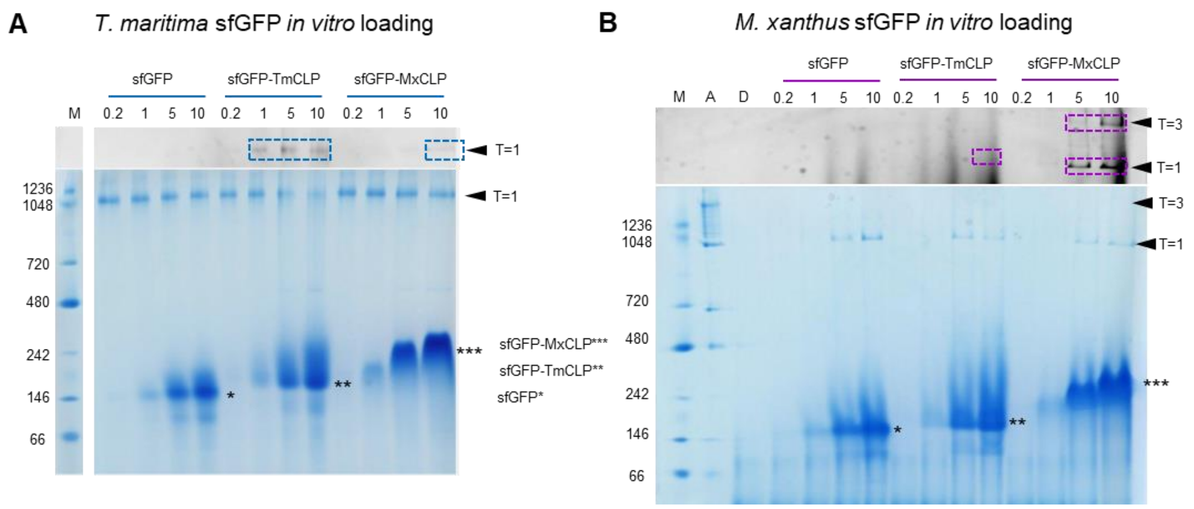

Figure S2: *In vitro* sfGFP loading is cargo loading peptide dependent. Molar ratio of sfGFP to encapsulin monomer 0.2, 1, 5 and 10 to 1 respectively. M=molecular weight marker. Top black and white image shows fluorescence signal of sfGFP. Bottom image shows Coomassie stained BN-PAGE gel. **A:** sfGFP loading into Tm_encap: No visible signal for sfGFP in fluorescence image, whereas fluorescence bands are visible with TmCLP and weak signal with MxCLP at 10:1. **B:** sfGFP loading into Mx_encap: No visible signal for sfGFP in fluorescence image, whereas fluorescence bands are visible with MxCLP and weak signal with TmCLP at 10:1.

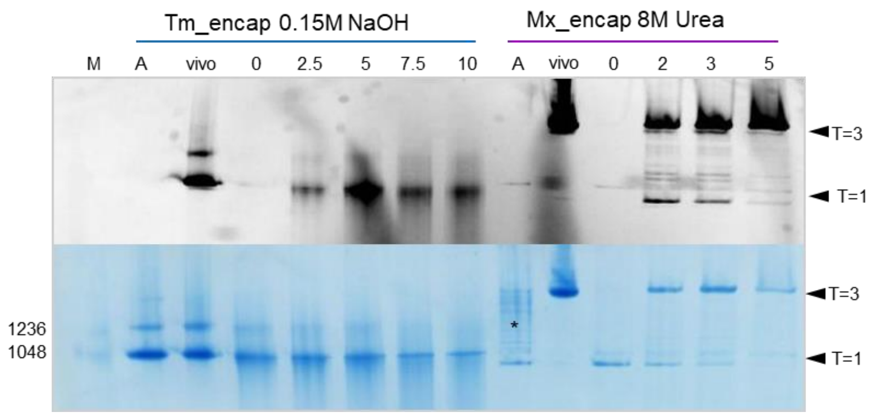

Figure S3: sfGFP cargo loading and scaffolding effect. sfGFP cargo loading into Tm_encap (disassembled in 0.15 M NaOH) and Mx_encap (disassembled in 8 M urea) at increasing concentration of sfGFP. Top black and white image shows fluorescence signal of sfGFP, bottom image shows Coomassie stained BN-PAGE gel. M=molecular weight marker, A=assembled (before denaturation), vivo=*in vivo* loaded encapsulins, numbers in lanes indicate molar ratio of sfGFP to encapsulin monomer. Asterisk indicates intermediate species between T=1 and T=3 capsids.

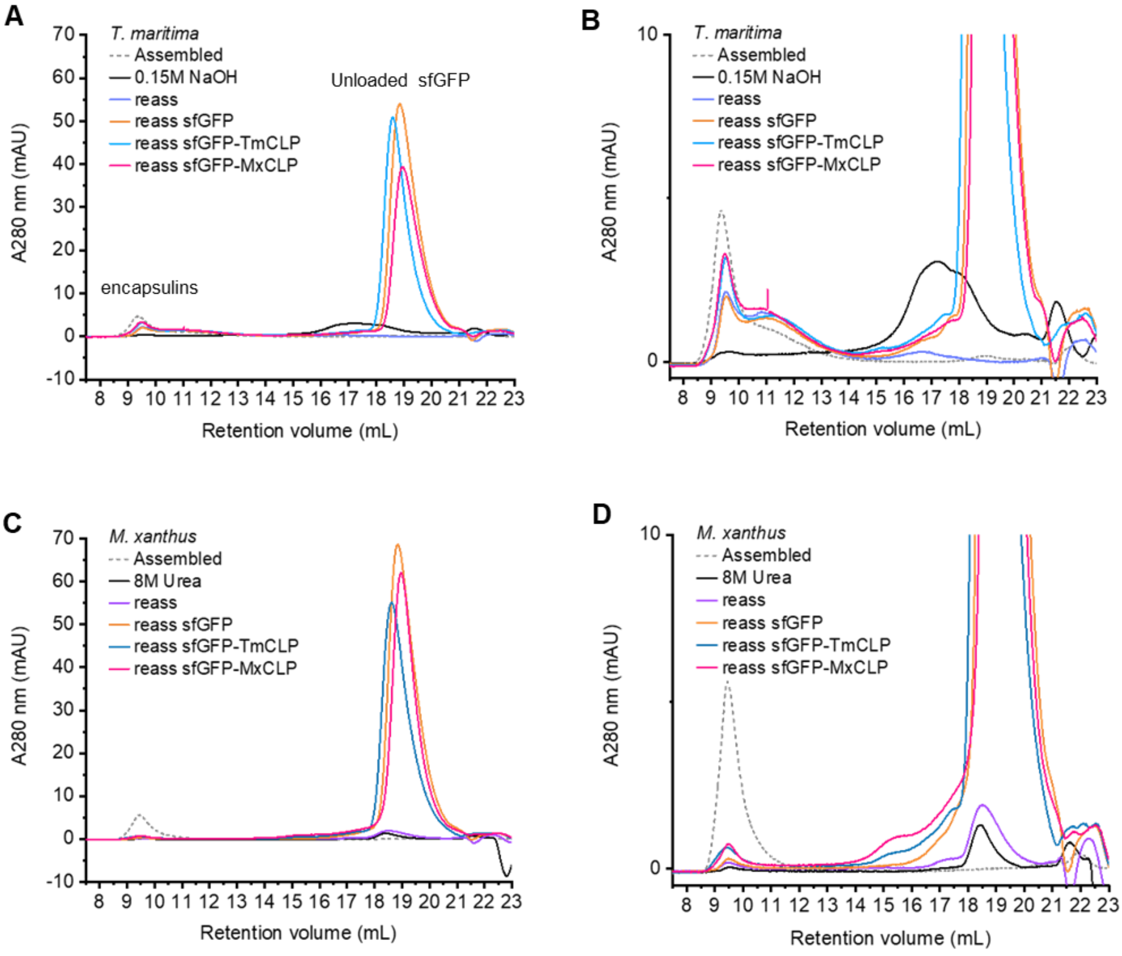

Figure S4: Size Exclusion Chromatography (SEC) profiles of sfGFP *in vitro* loading. **A-B:** B is a zoom-in on A. Tm_encap *in vivo* assembled and purified capsid (dashed line), following 0.15 M NaOH disassembly (black), reassembled in the absence of sfGFP (dark blue) and in the presence of sfGFP cargo at 5:1 molar ratio with TmCLP (light blue), MxCLP (red) and without CLP (yellow). **C-D:** D is a zoom-in on C. Mx_encap *in vivo* assembled and purified capsid (dashed line), following 8M Urea disassembly (black), assembled in the absence of sfGFP (purple), reassembly in the presence of sfGFP cargo at 5:1 molar ratio with TmCLP (light blue), MxCLP (red) and without CLP (yellow).

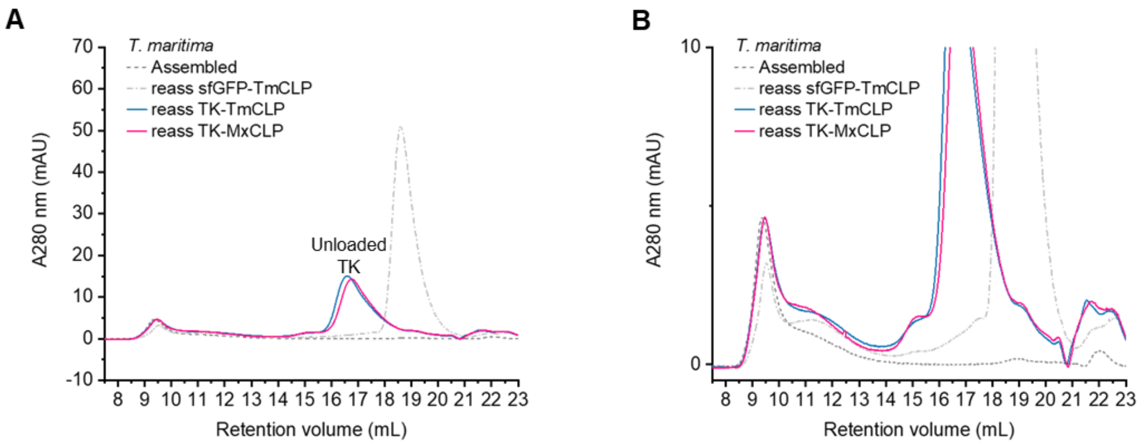

Figure S5: SEC profiles of transketolase *in vitro* loading. **A-B:** B is a zoom-in on A. Tm_encap reassembly with TK cargo at a 1:1 molar ratio (following 0.15 M NaOH disassembly). Tm_encap *in vivo* assembled and purified (dashed line), reassembled in the presence of TK-TmCLP (light blue), TK-MxCLP (red) and sfGFP-TmCLP as a reference (light grey dot-dash).

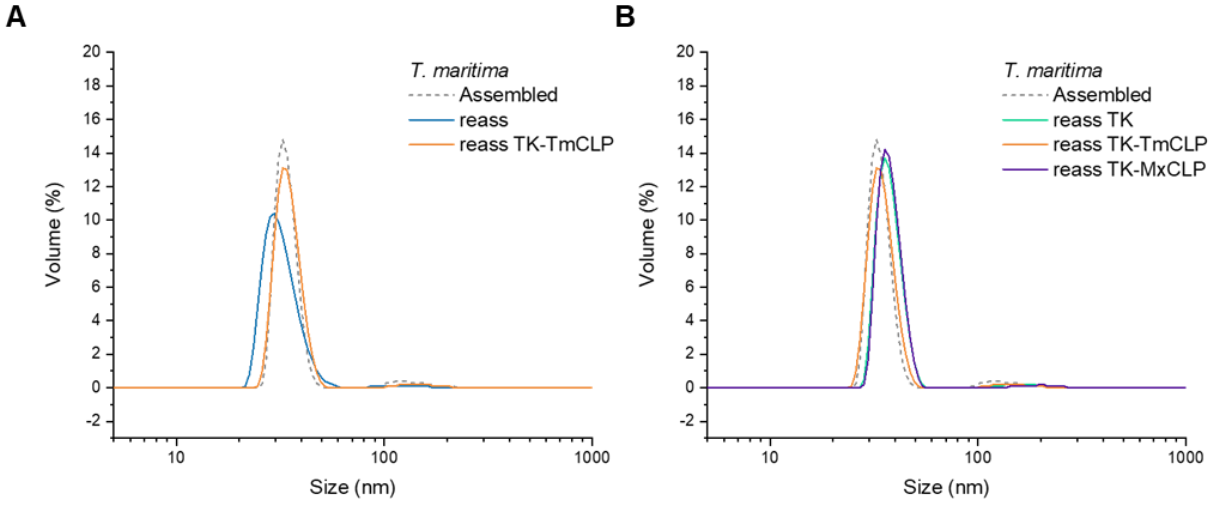

Figure S6: Dynamic Light Scatter (DLS) size frequency distributions by volume of reassembled Tm_encap. **A**: Tm_encap *in vivo* assembled and purified capsid (dashed line), reassembled following 0.15 M NaOH treatment in the absence of cargo (blue) and in the presence of TK-TmCLP cargo (orange) at 1:1 molar ratio. **B:** Tm_encap loading with TK with different CLPs. Tm_encap *in vivo* assembled and purified (dashed line), reassembled following 0.15 M NaOH treatment in the presence of TK without CLP (green), with TK-TmCLP (orange), TK-MxCLP (purple).

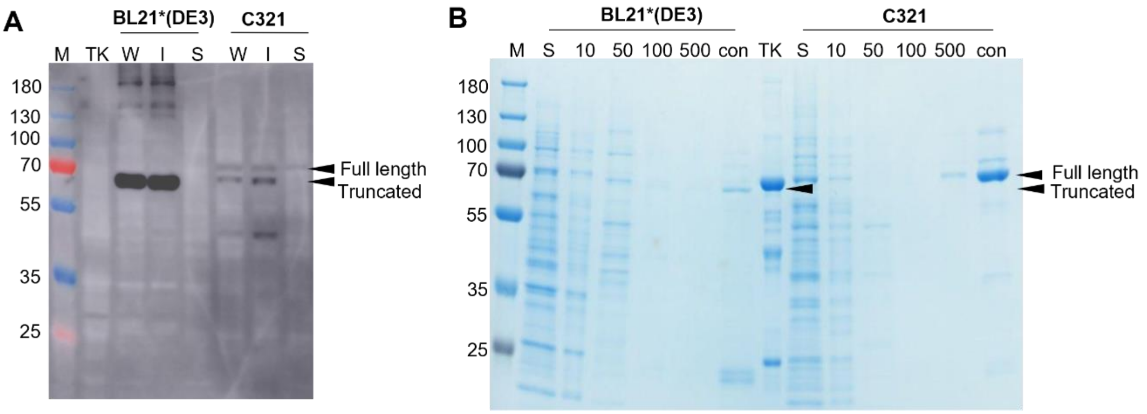

Figure S7: Expression TK-TmCLP in BL21Star (DE3) and C321 ΔA *E. coli* for pAzF incorporation. **A:** Anti-His Western blot of expression of TK-TmCLP K603pAzF in BL21 Star (DE3) and C321 strains. W=whole cell lysate, I=insoluble fraction and S=soluble fraction of lysate. TK=Transketolase without TmCLP and pAzF as control. Note, the anti-His antibody did not detect TK well. **B:** SDS-PAGE of purification of TK-TmCLP K603pAzF in BL21 Star (DE3) and C321 strains. S=Soluble lysate and IMAC fractions at increasing imidazole concentrations (mM), elution at 500 mM and 5-fold concentrated sample (con) of 500 mM elution fraction. TK=TK-TmCLP without pAzF as control.

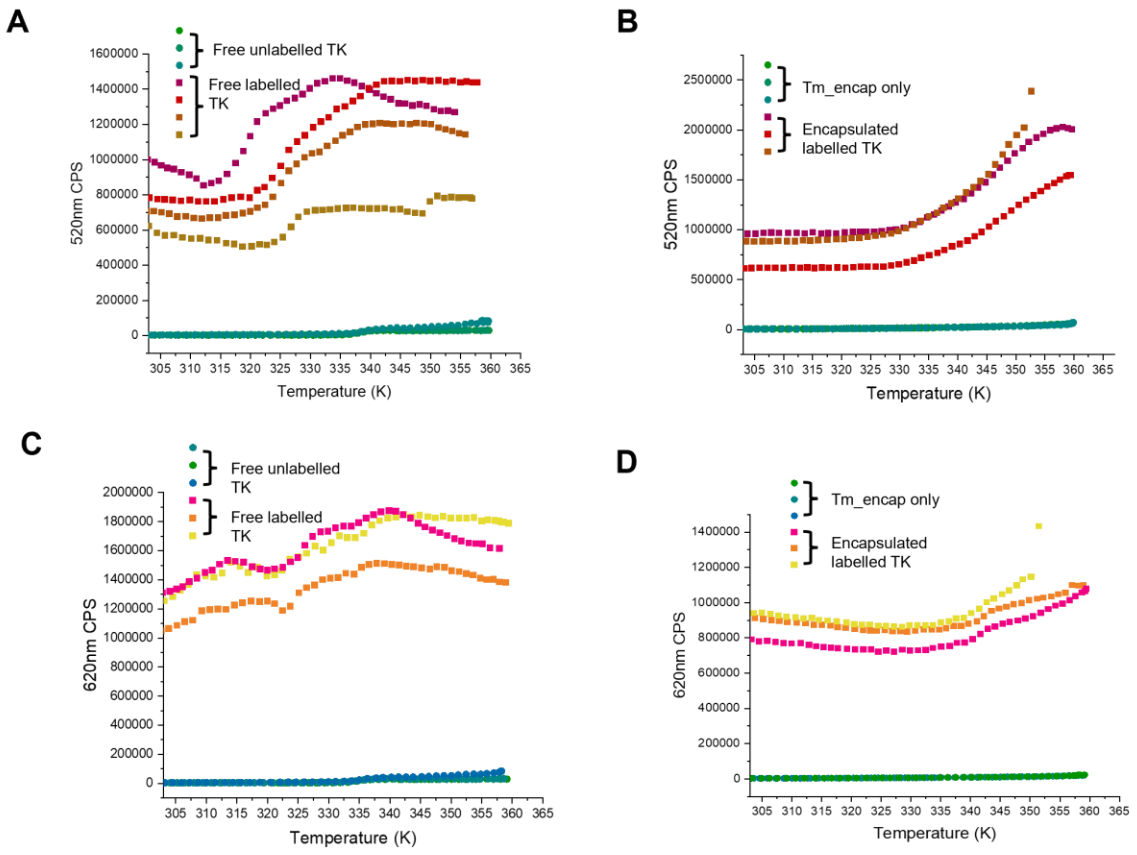

Figure S8: Raw local FRET denaturation donor and acceptor signal development of free and encapsulated TK. **A-B: FRET donor emission signal at 520 nm** of free unlabelled and AF488/594-labelled TK-TmCLP K603pAzF (**A**), and encapsulated AF488/594-labelled TK-TmCLP K603pAzF and empty reassembled Tm_encap as control (**B**). **C-D FRET acceptor emission signal at 620nm** of free unlabelled and AF488/594-labelled TK-TmCLP K603pAzF (**C**), and encapsulated AF488/594-labelled TK-TmCLP K603pAzF and empty reassembled Tm_encap as control (**D**). Free unlabelled and labelled TK-TmCLP K603pAzF are both at 0.1 mg/mL. Tm_encap at 0.04 mg/mL and encapsulated AF488/594-labelled TK-TmCLP K603pAzF at 0.008 mg/mL determined by SDS-PAGE densitometry. Different coloured data points indicate individual repeats. CPS=Raw photon Counts Per Second.

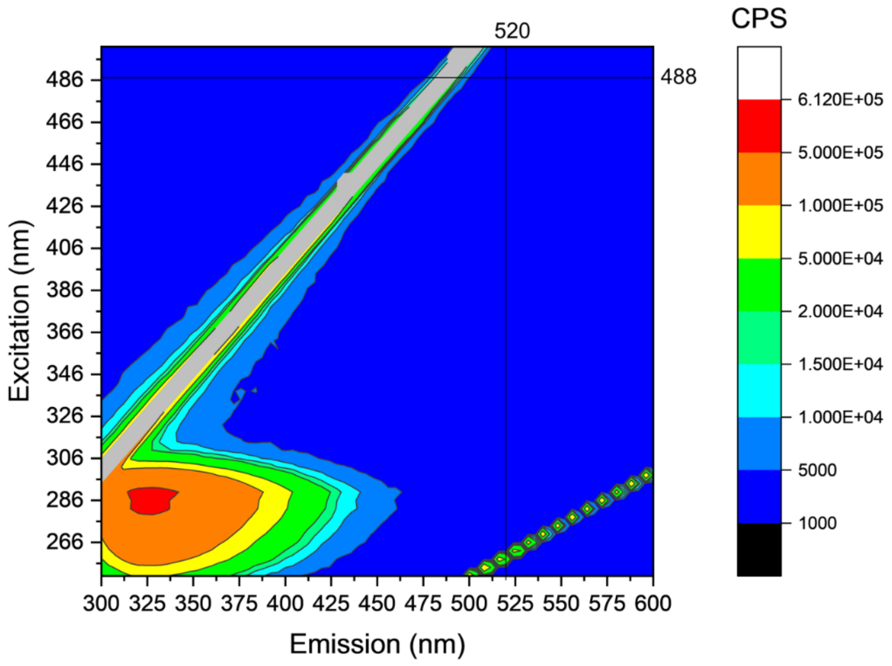

Figure S9: Excitation and emission 3D scan of Tm_encap at 0.4 mg/mL. Contour levels indicate Counts per Second (CPS) ranges given in the heat scale. Grey indicates incident light detection. The black drop lines indicate excitation and emission peak of FRET Donor AlexaFluor 488.

Table S1: Primers used for the addition of cargo loading peptides and site directed mutagenesis (SDM) of TK-TmCLP K603 residue.

Description	Name	Sequence 5' > 3'	Length (bp)
Tm_encap Cargo Loading Peptide oligos	Fw_TmCLPoligo_SacI_SalI	CATGAGCTCGGCAGCGGCGGCAGCGAAAACACCGGCGGCGATCTGGGCATTCGCAAACTGTAAGTCGACCGG	72
	Rv_TmCLPoligo_SacI_SalI	CCGGTCGACTTACAGTTTGCGAATGCCCAGATCGCCGCCGGTGTTTTCGCTGCCGCCGCTGCCGAGCTCATG	72
Mx_encap Cargo Loading Peptide oligos	Fw_mTPoligo_SacI_SalI	CGGCTCTGGCCTGACCGTGGGCAGCCTGCGCCGCTAAG	38
	Rv_mTPoligo_SacI_SalI	TCGACTTAGCGGCGCAGGCTGCCCACGGTCAGGCCAGAGCCGAGCT	46
SDM TK_K603TAG primers	Fw_TK_K603AMBR	GTACTGCCGTAGGCGGTTACTGCACGC	27
	Rv_TK_K603AMBR	GGATTCACGGTAAGCAGCATCCTGC	25

Table S2: List of strains and plasmids used in this study.

Name	Description	Source
C321 *ΔA exp*	'Amberless' E. coli strain, all genomic UAG Amber codons replaced with UAA and the Amber associated release factor (RF1) gene deleted. For Non-canonical Amino Acid (NcAA) incorporation.	Addgene plasmid # 49018
pET3a-Mx_encap_STII	*M. xanthus* encapsulin with C-terminal Strep-II tag	This study
pSB1C3-Tm_encap_STII	*T. maritima* encapsulin with C-terminal Strep-II tag	(28)
pJL1-sfGFP-MxCLP_Mx_encap_STII	*In vivo* loaded control: Mx_encap with sfGFP-MxCLP	This study
pJL1-sfGFP-TmCLP_Tm_encap_STII	*In vivo* loaded control: Tm_encap with sfGFP-TmCLP	This study
pJL1-HH_sfGFP	sfGFP with N-terminal Hexa-histidine tag	This study
pJL1-HH_sfGFP-MxCLP	sfGFP with N-terminal Hexa-histidine tag and C-terminal MxCLP	This study
pJL1-HH_sfGFP-TmCLP	sfGFP with N-terminal Hexa-histidine tag and C-terminal TmCLP	This study
pQR1623_HH-TK	Transketolase with N-terminal Hexa-histidine tag	This study
pQR1623_HH-TK-TmCLP	Transketolase with N-terminal Hexa-histidine tag and C-terminal TmCLP	This study
pQR1623_HH_TK-K603(TAG)-TmCLP	Transketolase mutated at position 603 from K to the Amber stop codon (TAG), with N-terminal Hexa-histidine tag and C-terminal TmCLP	This study
pULTRA-CNF	Plasmid containing the aminoacyl tRNA synthetase gene and tRNA gene. Used for Non-canonical Amino Acid (NcAA) incorporation in chemical competent C321 ΔA 'Amberless' *E. coli* strain.	Addgene plasmid #48215

DNA and amino acid sequences used in this study.

M. xanthus **encapsulin protein with C-terminal Strep-II tag (Mx_encap-STII):**

MPDFLGHAENPLREEEWARLNETVIQVARRSLVGRRILDIYGPLGAGVQTVPYDEFQGVSPGAVDIVGEQETAMV
FTDARKFKTIPIIYKDFLLHWRDIEAARTHNMPLDVSAAAGAAALCAQQEDELIFYGDARLGYEGLMTANGRLTVPL
GDWTSPGGGFQAIVEATRKLNEQGHFGPYAVVLSPRLYSQLHRIYEKTGVLEIETIRQLASDGVYQSNRLRGESGVV
VSTGRENMDLAVSMDMVAAYLGASRMNHPFRVLEALLLRIKHPDAICTLEGAGATERRTSGSGWSHPQFEK*

atgccggattttctgggccatgcggaaaacccgctgcgcgaagaagaatgggcgcgcctgaacgaaaccgtgattcaggtggcgcgccgcagc
ctggtgggccgccgcattctggatatttatggcccgctgggcgcgggcgtgcagaccgtgccgtatgatgaatttcagggcgtgagcccgggcgc
ggtggatattgtgggcgaacaggaaaccgcgatggtgtttaccgatgcgcgcaaatttaaaaccattccgattatttataaagattttctgctgca
ttggcgcgatattgaagcggcgcgcacccataacatgccgctggatgtgagcgcggcggcgggcgcggcggcgctgtgcgcgcagcaggaag
atgaactgatttttatggcgatgcgcgcctgggctatgaaggcctgatgaccgcgaacggccgcctgaccgtgccgctgggcgattggaccagc
ccgggcggcggctttcaggcgattgtggaagcgacccgcaaactgaacgaacagggccattttggcccgtatgcggtggtgctgagcccgcgcc
tgtatagccagctgcatcgcatttatgaaaaaaccggcgtgctggaaattgaaaccattcgccagctggcgagcgatggcgtgtatcagagcaa
ccgcctgcgcggcgaaagcggcgtggtggtgagcaccggccgcgaaaacatggatctggcggtgagcatggatatggtggcggcgtatctggg
cgcgagccgcatgaaccatccgtttcgcgtgctggaagcgctgctgctgcgcattaaacatccggatgcgatttgcaccctggaaggcgcgggcg
cgaccgaacgccgcactagtggcagcggctggagccacccgcagttcgaaaaataa

T. maritima **encapsulin protein with C-terminal enterokinase site and Strep-II tag (Tm_encap-STII):**

MSEFLKRSFAPLTEKQWQEIDNRAREIFKTQLYGRKFVDVEGPYGWEYAAHPLGEVEVLSDENEVVKWGLRKSLPL
IELRATFTLDLWELDNLERGKPNVDLSSLEETVRKVAEFEDEVIFRGCEKSGVKGLLSFEERKIECGSTPKDLLEAIVRA
LSIFSKDGIEGPYTLVINTDRWINFLKEEAGHYPLEKRVEECLRGGKIITTPRIEDALVVSERGGDFKLILGQDLSIGYED
REKDAVRLFITETFTFQVVNPEALILLKFSGASDDDDKGAWSHPQFEKTG*

atgtccgagtttctgaaacgcagcttcgccccgctgaccgagaagcagtggcaggagatcgacaatcgcgcccgcgagatcttcaagacacagc
tgtacggtcgcaagttcgtggacgtggaaggcccgtacggctgggaatatgccgcacaccctctgggtgaggtggaggtgctgagcgacgaga
acgaagtggttaagtggggtctgcgcaagagcctgccgttaatcgaactgcgcgcaaccttcaccctggacctgtgggagctggacaacctgga
gcgcggcaagccgaacgtggacctgagtagcctggaggaaaccgtgcgtaaggtggccgagtttgaggacgaagtgattttccgcggctgcga
gaagagcggcgttaagggtctgctgagcttcgaagagcgcaagatcgagtgcggcagcaccccgaaagatctgctggaggccatcgttcgcgc
cctgagcatcttcagtaaggacggcatcgagggcccgtacaccctggtgattaacaccgaccgttggatcaacttcctgaaagaagaggcgggt
cactacccgctggaaaaacgcgtggaagagtgtctgcgcggcggcaagatcatcaccacacctcgcatcgaagacgccttagtggttagcgag
cgcggcggcgactttaagctgatcctgggccaggacctgagcatcggctatgaggaccgtgaaaaggacgccgtgcgtctgttcatcacagaaa
ccttcaccttccaggtggtgaacccggaagccctgatcctgctgaagttcagcggtgcaagcgatgacgacgacaagggtgcatggagccaccc
gcagttcgaaaaaaccggttaa

sfGFP with N-terminal Hexa-histidine tag (HH-sfGFP):

MHHHHHHGSGMSKGEELFTGVVPILVELDGDVNGHKFSVRGEGEGDATIGKLTLKFICTTGKLPVPWPTLVTTLTY
GVQCFSRYPDHMKRHDFFKSAMPEGYVQERTISFKDDGKYKTRAVVKFEGDTLVNRIELKGTDFKEDGNILGHKLE
YNFNSHNVYITADKQKNGIKANFTVRHNVEDGSVQLADHYQQNTPIGDGPVLLPDNHYLSTQTVLSKDPNEKGTR
DHMVLHEYVNAAGITEL*

atgcatcatcaccatcaccacggaagcggcatgagcaaaggtgaagaactgtttaccggcgttgtgccgattctggtggaactggatggcgatg
tgaacggtcacaaattcagcgtgcgtggtgaaggtgaaggcgatgccacgattggcaaactgacgctgaaatttatctgcaccaccggcaaact
gccggtgccgtggccgacgctggtgaccaccctgacctatggcgttcagtgttttagtcgctatccggatcacatgaaacgtcacgatttctttaaa
tctgcaatgccggaaggctatgtgcaggaacgtacgattagctttaaagatgatggcaaatataaaacgcgcgccgttgtgaaatttgaaggcg

ataccctggtgaaccgcattgaactgaaaggcacggattttaaagaagatggcaatatcctgggccataaactggaatacaactttaatagcca
taatgtttatattacggcggataaacagaaaaatggcatcaaagcgaattttaccgttcgccataacgttgaagatggcagtgtgcagctggcag
atcattatcagcagaataccccgattggtgatggtccggtgctgctgccggataatcattatctgagcacgcagaccgttctgtctaaagatccga
acgaaaaaggcacgcgggaccacatggttctgcacgaatatgtgaatgcggcaggtattacggagctc

sfGFP with N-terminal Hexa-histidine tag with C-terminal *T. maritima* encapsulin Cargo loading peptide (HH-sfGFP-TmCLP):

MHHHHHHGSGMSKGEELFTGVVPILVELDGDVNGHKFSVRGEGEGDATIGKLTLKFICTTGKLPVPWPTLVTTLTY
GVQCFSRYPDHMKRHDFFKSAMPEGYVQERTISFKDDGKYKTRAVVKFEGDTLVNRIELKGTDFKEDGNILGHKLE
YNFNSHNVYITADKQKNGIKANFTVRHNVEDGSVQLADHYQQNTPIGDGPVLLPDNHYLSTQTVLSKDPNEKGTR
DHMVLHEYVNAAGITELGSGGSENTGGDLGIRKL*

atgcatcatcaccatcaccacggaagcggcatgagcaaaggtgaagaactgtttaccggcgttgtgccgattctggtggaactggatggcgatg
tgaacggtcacaaattcagcgtgcgtggtgaaggtgaaggcgatgccacgattggcaaactgacgctgaaatttatctgcaccaccggcaaact
gccggtgccgtggccgacgctggtgaccaccctgacctatggcgttcagtgttttagtcgctatccggatcacatgaaacgtcacgatttctttaaa
tctgcaatgccggaaggctatgtgcaggaacgtacgattagctttaaagatgatggcaaatataaaacgcgcgccgttgtgaaatttgaaggcg
ataccctggtgaaccgcattgaactgaaaggcacggattttaaagaagatggcaatatcctgggccataaactggaatacaactttaatagcca
taatgtttatattacggcggataaacagaaaaatggcatcaaagcgaattttaccgttcgccataacgttgaagatggcagtgtgcagctggcag
atcattatcagcagaataccccgattggtgatggtccggtgctgctgccggataatcattatctgagcacgcagaccgttctgtctaaagatccga
acgaaaaaggcacgcgggaccacatggttctgcacgaatatgtgaatgcggcaggtattacggagctcggcagcggcggcagcgaaaacacc
ggcggcgatctgggcattcgcaaactgtaa

sfGFP with N-terminal Hexa-histidine tag with C-terminal *M. xanthus* encapsulin Cargo loading peptide (HH-sfGFP-MxCLP) :

MHHHHHHGSGMSKGEELFTGVVPILVELDGDVNGHKFSVRGEGEGDATIGKLTLKFICTTGKLPVPWPTLVTTLTY
GVQCFSRYPDHMKRHDFFKSAMPEGYVQERTISFKDDGKYKTRAVVKFEGDTLVNRIELKGTDFKEDGNILGHKLE
YNFNSHNVYITADKQKNGIKANFTVRHNVEDGSVQLADHYQQNTPIGDGPVLLPDNHYLSTQTVLSKDPNEKGTR
DHMVLHEYVNAAGITELGSGLTVGSLRR*

atgcatcatcaccatcaccacggaagcggcatgagcaaaggtgaagaactgtttaccggcgttgtgccgattctggtggaactggatggcgatg
tgaacggtcacaaattcagcgtgcgtggtgaaggtgaaggcgatgccacgattggcaaactgacgctgaaatttatctgcaccaccggcaaact
gccggtgccgtggccgacgctggtgaccaccctgacctatggcgttcagtgttttagtcgctatccggatcacatgaaacgtcacgatttctttaaa
tctgcaatgccggaaggctatgtgcaggaacgtacgattagctttaaagatgatggcaaatataaaacgcgcgccgttgtgaaatttgaaggcg
ataccctggtgaaccgcattgaactgaaaggcacggattttaaagaagatggcaatatcctgggccataaactggaatacaactttaatagcca
taatgtttatattacggcggataaacagaaaaatggcatcaaagcgaattttaccgttcgccataacgttgaagatggcagtgtgcagctggcag
atcattatcagcagaataccccgattggtgatggtccggtgctgctgccggataatcattatctgagcacgcagaccgttctgtctaaagatccga
acgaaaaaggcacgcgggaccacatggttctgcacgaatatgtgaatgcggcaggtattacggagctcggctctggcctgaccgtgggcagcct
gcgccgctaa

Transketolase (TK) with N-terminal Hexa-histidine tag and C-terminal *T. maritima* CLP (HH-TK-TmCLP):

MHHHHHHSSRKELANAIRALSMDAVQKAKSGHPGAPMGMADIAEVLWRDFLKHNPQNPSWADRDRFVLSNG
HGSMLIYSLLHLTGYDLPMEELKNFRQLHSKTPGHPEVGYTAGVETTTGPLGQGIANAVGMAIAEKTLAAQFNRP
GHDIVDHYTYAFMGDGCMMEGISHEVCSLAGTLKLGKLIAFYDDNGISIDGHVEGWFTDDTAMRFEAYGWHVIR
DIDGHDAASIKRAVEEARAVTDKPSLLMCKTIIGFGSPNKAGTHDSHGAPLGDAEIALTREQLGWKYAPFEIPSEIYA
QWDAKEAGQAKESAWNEKFAAYAKAYPQEAAEFTRRMKGEMPSDFDAKAKEFIAKLQANPAKIASRKASQNAIE
AFGPLLPEFLGGSADLAPSNLTLWSGSKAINEDAAGNYIHYGVREFGMTAIANGISLHGGFLPYTSTFLMFVEYARN

AVRMAALMKQRQVMVYTHDSIGLGEDGPTHQPVEQVASLRVTPNMSTWRPCDQVESAVAWKYGVERQDGPT
ALILSRQNLAQQERTEEQLANIARGGYVLKDCAGQPELIFIATGSEVELAVAAYEKLTAEGVKARVVSMPSTDAFDK
QDAAYRESVLPKAVTARVAVEAGIADYWYKYVGLNGAIVGMTTFGESAPAELLFEEFGFTVDNVVAKAKELLGSG
GSENTGGDLGIRKL*

atgcatcaccatcaccatcactcctcacgtaaagagcttgccaatgctattcgtgcgctgagcatggacgcagtacagaaagccaaatccggtca
cccgggtgcccctatgggtatggctgacattgccgaagtcctgtggcgtgatttcctgaaacacaacccgcagaatccgtcctgggctgaccgtg
accgcttcgtgctgtccaacggccacggctccatgctgatctacagcctgctgcacctcaccggttacgatctgccgatggaagaactgaaaaac
ttccgtcagctgcactctaaaactccgggtcacccggaagtgggttacaccgctggtgtggaaaccaccaccggtccgctgggtcagggtattgc
caacgcagtcggtatggcgattgcagaaaaaacgctggcggcgcagtttaaccgtccgggccacgacattgtcgaccactacacctacgccttc
atgggcgacggctgcatgatggaaggcatctcccacgaagtttgctctctggcgggtacgctgaagctgggtaaactgattgcattctacgatga
caacggtatttctatcgatggtcacgttgaaggctggttcaccgacgacaccgcaatgcgtttcgaagcttacggctggcacgttattcgcgacat
cgacggtcatgacgcggcatctatcaaacgcgcagtagaagaagcgcgcgcagtgactgacaaaccttccctgctgatgtgcaaaaccatcatc
ggtttcggttccccgaacaaagccggtacccacgactcccacggtgcgccgctgggcgacgctgaaattgccctgacccgcgaacaactgggct
ggaaatatgcgccgttcgaaatcccgtctgaaatctatgctcagtgggatgcgaaagaagcaggccaggcgaaagaatccgcatggaacgag
aaattcgctgcttacgcgaaagcttatccgcaggaagccgctgaatttacccgccgtatgaaaggcgaaatgccgtctgacttcgacgctaaagc
gaaagagttcatcgctaaactgcaggctaatccggcgaaaatcgccagccgtaaagcgtctcagaatgctatcgaagcgttcggtccgctgttgc
cggaattcctcggcggttctgctgacctggcgccgtctaacctgaccctgtggtctggttctaaagcaatcaacgaagatgctgcgggtaactaca
tccactacggtgttcgcgagttcggtatgaccgcgattgctaacggtatctccctgcacggtggcttcctgccgtacacctccaccttcctgatgttc
gtggaatacgcacgtaacgccgtacgtatggctgcgctgatgaaacagcgtcaggtgatggtttacacccacgactccatcggtctgggcgaag
acggcccgactcaccagccggttgagcaggtcgcttctctgcgcgtaaccccgaacatgtctacatggcgtccgtgtgaccaggttgaatccgcg
gtcgcgtggaaatacggtgttgagcgtcaggacggcccgaccgcactgatcctctccaggcagaacctggcgcagcaggaacgaactgaagag
caactggcaaacatcgcgcgcggtggttatgtgctgaaagactgcgccggtcagccggaactgattttcatcgctaccggttcagaagttgaact
ggctgttgctgcctacgaaaaactgactgccgaaggcgtgaaagcgcgcgtggtgtccatgccgtctaccgacgcatttgacaagcaggatgct
gcttaccgtgaatccgtactgccgaaagcggttactgcacgcgttgctgtagaagcgggtattgctgactactggtacaagtatgttggcctgaac
ggtgctatcgtcggtatgaccaccttcggtgaatctgctccggcagagctgctgtttgaagagttcggcttcactgttgataacgttgttgcgaaag
caaaagaactgctgggctcgggcgggtctgaaaacactggaggcgatttgggcatccgcaaactgtaa
